# Novel Bromo and methoxy substituted Schiff base complexes of Mn(II), Fe(III), and Cr(III) for anticancer, antimicrobial, docking, and ADMET studies

**DOI:** 10.1038/s41598-023-29386-2

**Published:** 2023-02-23

**Authors:** Laila H. Abdel-Rahman, Amani A. Abdelghani, Abeer A. AlObaid, Doaa Abou El-ezz, Ismail Warad, Mohamed R. Shehata, Ehab M. Abdalla

**Affiliations:** 1 Chemistry Department, Faculty of Science, Sohag University, Sohag, 82534 Egypt; 2 Department of Chemistry, University of Prince Edward Island, 550 University Avenue, Charlottetown, PE C1A 4P3 Canada; 3 Chemistry Department, Faculty of Science, Damanhour University, Damanhour, 22511 Egypt; 4 Department of Chemistry, College of Science, King Saud University, Riyadh-11451, Saudi Arabia; 5 Department of Pharmacology and Toxicology, Faculty of Pharmacy, October University for Modern Sciences and Arts (MSA University), Cairo, Egypt; 6 Department of Chemistry, AN-Najah National University, P.O. Box 7, Nablus, Palestine; 7 Chemistry Department, Faculty of Science, Cairo University, Giza, Egypt; 8 Chemistry Department, Faculty of Science, New Valley University, Alkharga, 72511 Egypt

**Keywords:** Biological techniques, Biotechnology, Cancer, Chemical biology, Computational biology and bioinformatics, Developmental biology, Drug discovery, Microbiology, Chemistry, Mathematics and computing

## Abstract

In this study, four new Mn(II), Fe(III), and Cr(III) complexes with two Schiff base ligands namely, 4-bromo-2-[(E)-{[4-(2-hydroxyethyl)phenyl]imino}methyl]phenol (HL1) and 2-[(E)-{[4-(2-hydroxyethyl)phenyl]imino}methyl]-4-methoxy phenol (HL2) have been synthesized and characterized. Different analytical and spectral methods have been used to characterize the ligands and their complexes. General formulas of [M(L)Cl_2_(H_2_O)_2_] for FeL1, CrL1 and CrL2, and [M(L)Cl(H_2_O)_3_] for MnL2 were proposed. HOMO and LUMO energies, as well as the electrical characteristics, have been calculated using DFT/B3LYP calculations with Gaussian 09 program. The optimized lowest energy configurations of the complexes are proven. The disc diffusion technique was used to test the pharmacological activities' antibacterial efficacy against diverse bacterial and fungus species. The MTT technique was used to assess the in vitro cytotoxicity of the ligands and their metal complexes on the Hep-G2 human liver carcinoma cell line and the MCF-7 human breast cancer cell line. All compounds displayed better activity compared to the free ligands. MnL2 complex showed predominant activity when compared to the other complexes with an IC_50_ value of 2.6 ± 0.11 μg/ml against Hep-G2, and against MCF-7 the IC_50_ value was 3.0 ± 0.2 μg/ml which is less than the standard drug cisplatin (4.0 μg/ml). UV–vis electronic spectrum and gel electrophoresis techniques have been used to investigate the compounds’ affinity to bind and cleavage CT-DNA. The interaction’s binding constants, or Kb, have been identified, and it was discovered that the new complexes' binding affinities are in the order of FeL1 > MnL2 > CrL2 > CrL1, and the binding mechanism has been suggested. To assess the kind of binding and binding affinity of the investigated drugs with human DNA, a molecular docking study was carried out (PDB:1bna). The acquired results supported the intercalation binding mechanism proposed in the experimental part and revealed that complexes may be inserted into the DNA molecule to stop DNA replication. According to ADMET data, the synthesized compounds have a high bioavailability profile and their physicochemical and pharmacological features remained within Lipinski's RO5 predicted limitations.

## Introduction

In recent years, many researchers have focused on Schiff bases and related complexes as a significant class of therapeutic molecules with a vast potential for biological efficacies like antibacterial, anticancer, anticonvulsant, and antioxidant properties^1–9^. The salicylaldimine complexes are utilized as models for many theoretical interests and structural, magnetic, spectroscopic, catalytic, and redox characteristics^10–12^. There have been reports of the pharmacological properties of Schiff base complexes with NO donor atoms, including biological activity against bacteria, fungi, and specific types of malignancies^13,14^. Stable complexes of the NO bidentate Schiff base ligands containing the methoxy group formed through coordination with transition metal ions. The resulting complexes have a variety of uses, including catalysis, electrocatalysis, biochemistry, and spectroscopy^15,16^. It has shown that the imine group present in the bidentate ligands plays a critical role in their biological activities^17–20^. The coordination chemistry of transition metal complexes containing ligands of the salicylaldimine family that have been substituted is of considerable and rising interest. Non-platinum transition metal complexes have received great attention recently because they may be able to reduce the severe side effects and high cost of care associated with platinum-based medication prospects.

The manganese(II), iron(III), and chromium(III) Schiff base complexes of azomethine donors exhibit an important function in nucleic acid chemistry because of their structural stability and adaptability to biological proteins^21,22^. A substantial number of Schiff base manganese (II), iron (III), and chromium complexes are successful models of biological systems and have a potential biological interest^23^, such as supramolecular and helical assemblies^24^.

One of the major health crises of today’s world is antimicrobial drug resistance and there is a great need to discover new compounds that may be effective against antibiotic-resistant bacteria, given the increased occurrence of serious opportunistic bacterial infections in immunologically deficient patients, along with the growth of resistance among pathogenic gram-negative and gram-positive bacteria. Motivated by the promising structural and biological properties of Schiff based metal complexes together with our perpetual engagement in this kind of research endeavors^26–30^ and^32–35^, we offer here facile approaches for the synthesis and characterization of two novel bidentate Schiff base ligands (HL1 and HL2), (4-bromo-2-[(E)-{[4-(2-hydroxyethyl)phenyl]imino}methyl]phenol (HL1) and 2-[(E)-{[4-(2-hydroxyethyl)phenyl]imino}methyl]-4-methoxy phenol (HL2)) and their metal complexes CrL1, FeL1, CrL2 and MnL2. Various spectroscopic, structural, and analytical techniques combined with theoretical DFT calculations have been employed to deduce the composition, structure, and geometry of the prepared complexes. The pharmacological activities’ antibacterial efficacy against diverse bacterial and fungus species. The MTT technique was used to assess the in vitro cytotoxicity of the ligands and their metal complexes on the Hep-G2 human liver carcinoma cell line and the MCF-7 human breast cancer cell line. Additionally, as CT-DNA is considered a primary target molecule for the majority of anti-cancer and antiviral medicines, the binding manner of the metal azomethine chelates with CT-DNA will be suggested. Moreover, to assess the kind of binding and binding affinity of the investigated drugs with human DNA, a molecular docking study was carried out (PDB:1bna). The pharmacokinetic and biological actions of the two Schiff bases and their complexes were predicted using Swiss ADME, PASS and pkCSM online software.

## Experimental

### Materials

All of the substances utilized, including 2-(4-aminophenyl)ethan-1-ol amine, 5-bromosalicylaldehyde, 5-methoxysalicylaldehyde, CrCl_3_.6H_2_O, FeCl_3_.6H_2_O and MnCl_2_.4H_2_O were of analytical quality and were acquired from Sigma Aldrich.

Supplementary file (Section S1) comprises detailed information of the instruments and methods utilized for structure confirmation and application***.***

### Synthesis

#### Synthesis of the ligands

**Scheme 1:**
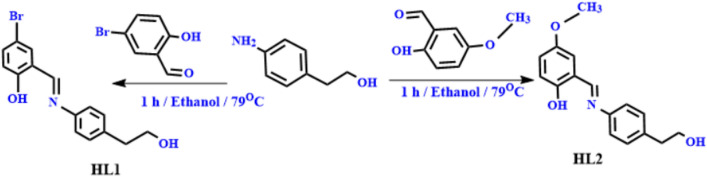
Synthesis of the novel bromo Schiff base HL1 and methoxy Schiff base HL2.

Condensation of 2-(4-aminophenyl)ethan-1-ol (1 mmol, 0.14 gm), which was dissolved in ethyl alcohol, was used to prepare the ligands (20 ml). The product solution was diluted by 20 ml, and either 1 mmol of 5-methoxy salicylaldehyde (0.30 gm) or 1 mmol of 5-bromo salicylaldehyde (0.20 gm) was to it. The final mixture was refluxed at 79 °C for one hour. Orange precipitate was collected, filtered, and subjected to several ethanol washes. The mixtures were vacuum-dried over anhydrous CaCl_2_. HL1 had a yield percentage of 78%, whereas HL2 had a yield rate of 79%. (Scheme 1).

#### Synthesis of complexes

20 ml of EtOH were used to dissolve a 1 mmol solution of the various metal salts [CrCl_3_.6H_2_O, 0.26 g), (FeCl_3_.6H_2_O, 0.27 g) or (MnCl_2_._4_H_2_O, 0.12 g)]. The metal ion solution was dropwise mixed with a 1 mmol solution of the bidentate ligand, and the solution was refluxed for 2 to 6 h at 79 °C while being stirred. The precipitate that resulted from this was gathered using a filter, washed with ethanol, and afterward dried in an oven^23^. (Structure 1).

**Structure 1:**
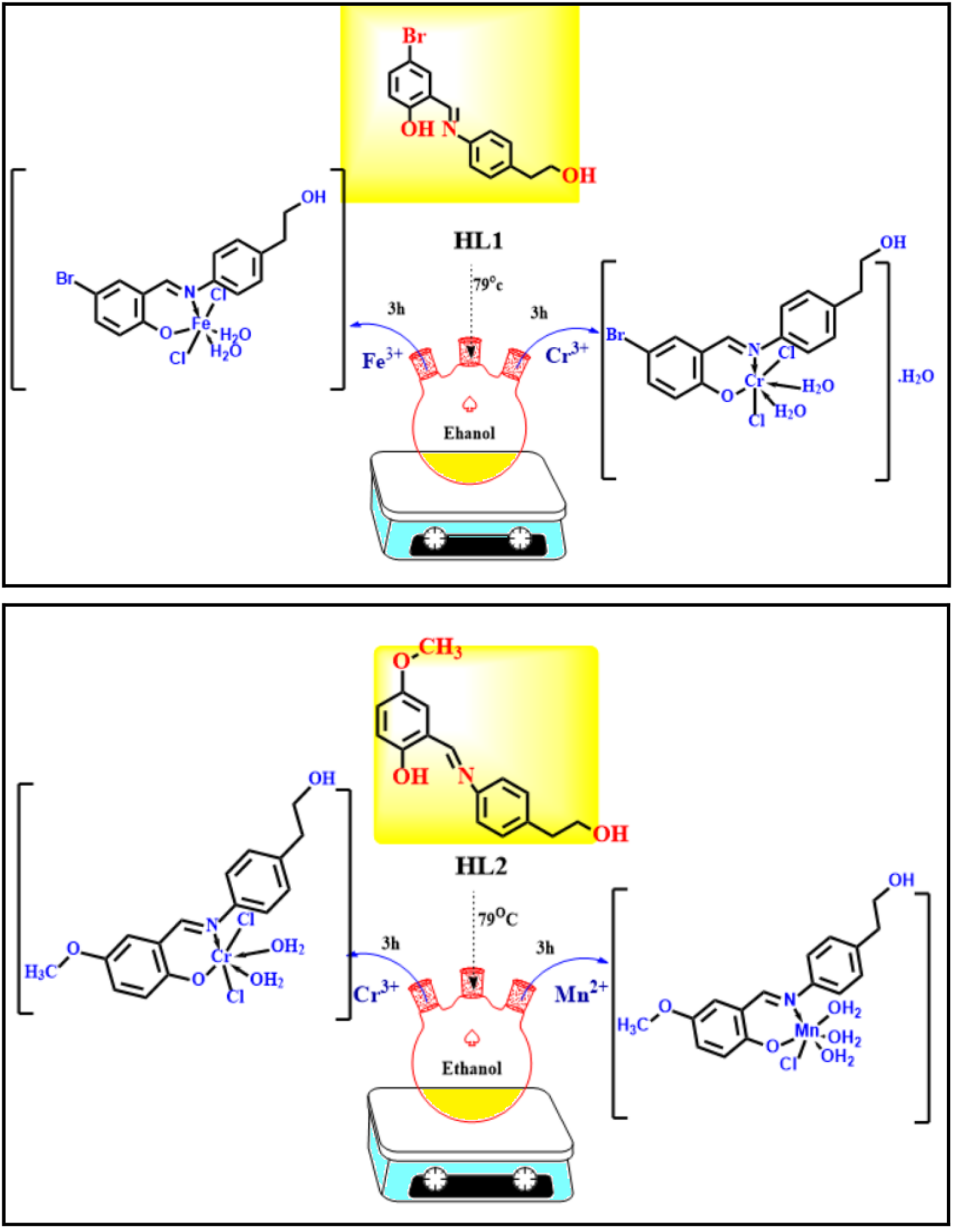
The synthesis of the novel HL1, HL2 ligands, their metal complexes and their predicted structures.

### Theoretical DFT studies

We used the Gaussian 09 computer program to conduct a Density Functional Theory (DFT) analysis to ascertain the optimal structures of the ligands HL1, HL2, and their complexes^24^. For the trivalent metals Cr(III), Fe(III), and bivalent Mn(II), we employed the hybrid exchange–correlation function with the basis set levels of B3LYP/6-31G (d, p) for C, N, and O and B3LYP/LANL2DZ for trivalent metals Cr(III), Fe(III), and bivalent Mn(II).

### Biological activity

#### Antimicrobial activity

Using the Petri dish methodology, the newly synthesized compounds were tested against various species of bacteria. Staphylococci aureus ATCC 25923, Escherichia coli ATCC 25922, Bacillus subtilis RCMB 015(1) NRRLB-543, Pseudomonas vulgaris RCMB 004 (1) ATCC 13315, A. Albicans RCMB005003 (1) ATCC 10231, and A. Fumigatus (RCMB 002008) are among the bacterial species. At different dosages, such as 10, 20, 50, and 100 g/ml, the antibacterial and antifungal activity of the two ligands, as well as their CrL1, FeL1, CrL1, and MnL2 complexes, was assessed^25^. In the supplemental file, specifics of the antimicrobial research' methodology are illustrated (Section S2).

#### Cytotoxicity

The MTT [3(4,5-dimethylthiazol-2-yl)-2,5-diphenyltetrazolium bromide] test was performed against the Hep-G2 liver carcinoma cell line and the MCF7 breast cancer cell line to determine the cytotoxicity of the two ligands and their Mn(II), Fe(III), and Cr(III) complexes. Each well of the 96-well plate was seeded with 5000 cells. Using the complete medium, the cell suspension was diluted to a concentration of 5 × 10^4^ cells/ml. Each well received 100 ml of the cell solution pipetted into it using a micropipette. For 24 h, 37 °C was maintained on the plate to encourage cell attachment.

Following the synthesis of novel compounds, 100 ml of growth media containing 0, 0.001, 0.01, 0.1, 1, and 100 l of each medication were used to treat cells in triplicate^25^. Other details are in the supplementary file (Section S3).

#### CT-DNA binding experiment

A 260/280 UV absorbance ratio of 1.9 was found for CT-DNA in a NaCl/HCl buffer with a pH of 7.2, indicating that it was protein-free^27^. To create the stock DNA, the regular use of sonication was performed throughout 25 cycles with 1-minute breaks at 4 °C.

##### Absorption spectral studies

The novel compounds’ affinity for interacting with the DNA of the calf thymus was examined using absorption spectra (CT-DNA). Electronic absorption spectra are acquired utilizing a 1.0 cm quartz cuvette at room temperature, keeping the complex concentration at (1 × 10^−3^ mol L^−1^) and progressively raising the calf thymus DNA concentration from (10 μM) to (100 μM). The interactions of the new Fe(III), Cr(III), and Mn(II) complexes with calf thymus DNA were calculated using the equation shown below to determine their intrinsic binding constant (Kb)^26^:

1 $${\raise0.7ex\hbox{${\left[ {{\text{DNA}}} \right]}$} \!\mathord{\left/ {\vphantom {{\left[ {{\text{DNA}}} \right]} {\left( {{\upvarepsilon }_{{\text{a}}} - {\upvarepsilon }_{{\text{f}}} } \right)}}}\right.\kern-0pt} \!\lower0.7ex\hbox{${\left( {{\upvarepsilon }_{{\text{a}}} - {\upvarepsilon }_{{\text{f}}} } \right)}$}} = \left[ {{\text{DNA}}} \right]\frac{1}{{\left( {{\upvarepsilon }_{{\text{b}}} - {\upvarepsilon }_{{\text{f}}} } \right)}} + \frac{1}{{{\text{K}}_{{\text{b}}} }}\frac{1}{{\left[ {\left( {{\upvarepsilon }_{{\text{b}}} - {\upvarepsilon }_{{\text{f}}} } \right)} \right]}}$$

where:

${\upvarepsilon }_{\mathrm{a}}$ is used to describe the apparent absorption coefficients for free DNA.

${\upvarepsilon }_{\mathrm{f}}$ is used to describe the apparent absorption coefficients for the free metal complex.

${\upvarepsilon }_{\mathrm{b}}$ explains the apparent absorption coefficients for bounded metal complexes.

To evaluate the standard free energy of the DNA-binding fo, the following equation was utilized^27–29^:

2 $$\Delta {\text{G}}_{{\text{b}}}^{ \ne } = - {\text{RT linK}}_{{\text{b}}}$$

##### Agarose gel electrophoresis

Using agarose gel electrophoresis, our research has previously discussed how the unique Cr(III), Fe(III), and Mn(II) complexes interact with CT-DNA^24^.

#### Molecular docking

With the use of molecular docking, the stability of different metal Schiff base complexes can be examined, and the optimal binding alignment and shape for molecules interacting in space may be anticipated. The docking experiment was carried out on a Dell Precision TM T3600 workstation adopting the Molecular Operating Environment (MOE) package version 2016.08. We employed the 1.9 resolution X-ray crystal structure of a B-DNA dodecamer d(CGCGAATTCGCG)2 running in the 3'-5' direction for our docking experiment (PDB ID: 1BNA).The MOE software was used to evaluate the DNA structure before hydrogen atoms were added and an energy optimization procedure was carried out.

The generated model was put by a thorough conformational search using the default position finding tool parameters of the MOE program and an RMS gradient of 0.01 kcal/mol. The Schiff base ligands (HL1 and HL2) and their complexes with trivalent metals (Cr and Fe) and bivalent Mn were included in Chem Bio Draw Ultra 12.0 for further development in MOE. The docking procedures were followed exactly as described in the literature^30^.

## Results and discussion

The analytical outcomes for the HL1 and HL2 Schiff bases, as well as those of their complexes with Fe(III), Mn(II), and Cr(III), are all displayed in Table 1 and are in good agreement with the anticipated molecular formula for the complexes in Structure 1. Results from elemental investigations on the complexes of Cr(III), Fe(III), and Mn(II) show that they typically have the formula [M(L)Cl_2_(H_2_O)_2_] for FeL1, CrL1, and CrL2, and [M(L)Cl(H_2_O)_3_] for MnL2. A DMSO solution (1 × 10^−3^ M) is used to evaluate the molar conductance of metal complexes at ambient temperature. The results show that the metal complexes with molar conductance values of 2.68 to 8.12 S cm^2^ mol^−1^ are non-electrolytes.

**Table 1:** Data on the two ligands’ new Cr(III), Fe(III), and Mn(II) complexes from elemental analysis.

Compound	M. Wt	Λ m@ Ω^−1^ cm^2^ mol^−1^	(M. p)°C	μ_eff_ B.M	Found (calc.) %					
					C	H	N	Cl	Br	M
HL1	320.19	0.00	210	–	56.23 (56.27)	4.39 (4.41)	4.33 (4.37)	–	24.91 (24.96)	–
HL2	271.32	0.00	190	–	70.81 (70.83)	6.29 (6.32)	5.14 (5.16)	–	–	–
FeL1	481.95	5.69	> 300	5.19	38.34 (37.38)	3.52 (3.56)	2.87 (2.91)	14.63 (14.71)	16.51 (16.58)	11.56 (11.59)
CrL1	496.12	5.89	> 300	4.01	36.28 (36.31)	3.84 (3.86)	2.77 (2.82)	14.26 (14.29)	16.04 (16.11)	10.41 (10.48)
MnL2	432.76	2.68	> 300	5.22	44.37 (44.41)	5.56 (5.59)	3.20 (3.24)	8.13 (8.19)	–	12.67 (12.69)
CrL2	429.23	8.12	> 300	4.05	44.74 (44.77)	4.68 (4.70)	3.23 (3.26)	16.46 (16.52)	–	12.09 (12.11)

### ^1^HNMR spectra

**Figure 1:**
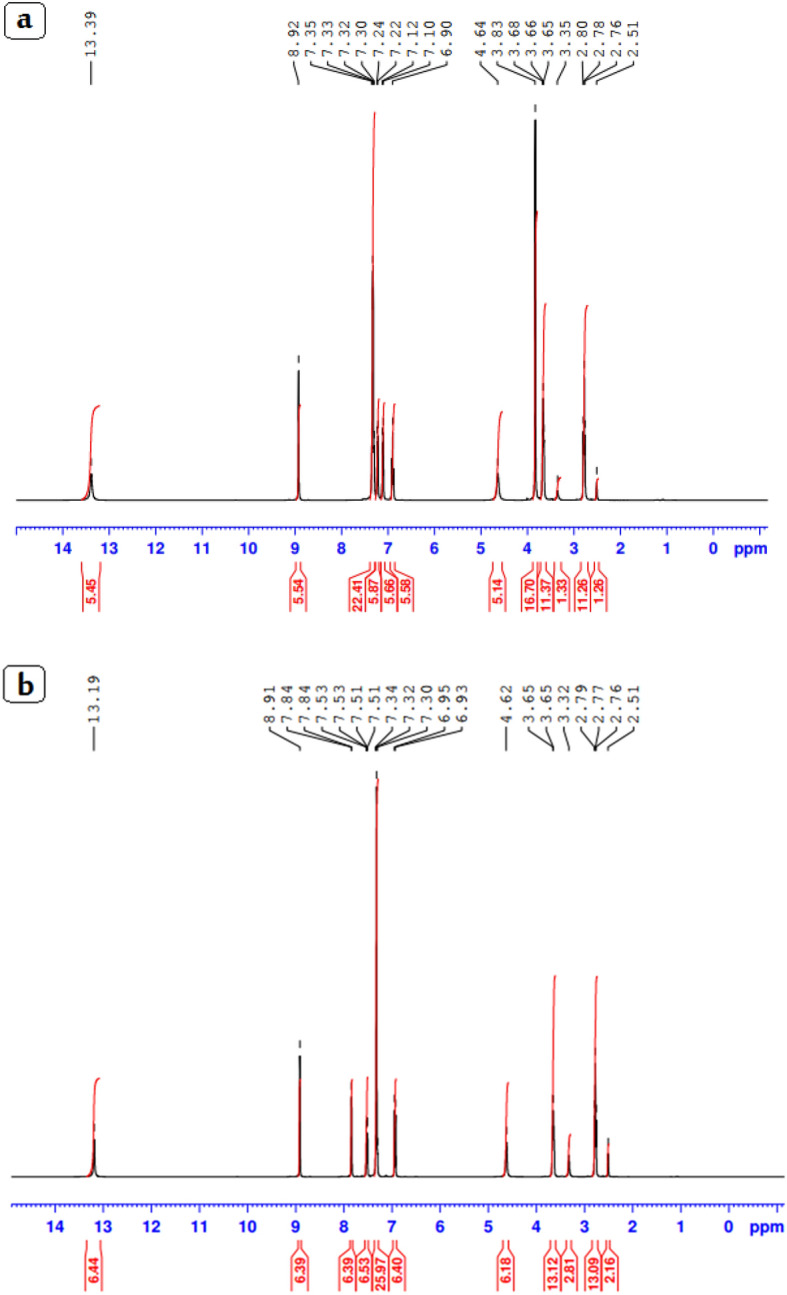
^1^HNMR spectra of HL1 (**a**) and HL2 ligands (**b**).

Figure 1 displays the ^1^HNMR spectra of the two ligands (a, b). The aromatic protons may be the origin of the multiplets that were seen in the spectra of both ligands, which were in the range of 6.90–7.35 and 6.90–7.35 ppm for ligand HL1 and HL2, respectively. The proton of the azomethine group (H, CH = N) is represented by the singlet signal at 8.91 ppm in the spectra of the two ligands, HL1 and HL2 (Scheme 1). The phenolic OH proton was found in HL1 and HL2 at 13.19 and 13.39 ppm, respectively.

The 4H of the ethyl group (CH_2_–CH_2_) of the side chains of the two ligands may be responsible for the multiplet signals for HL1 and HL2 occurring at 2.76–3.65 ppm and 2.51–3.83 ppm, respectively. The typical signal for the –CH = N group was visible at 161.53 and 163.17 ppm in the ^13^C NMR spectra for HL1 and HL2, respectively, in Figure S1. While signals appeared in the region 119.46–159.87 and 116.10–151.21 ppm for aromatic carbon atoms of the ligands HL1 and HL2.

### Infrared spectral analysis

The related Cr(III), Mn(II), and Fe(III) complexes, as well as the ligands HL1 and HL2, have FTIR spectra that are shown in Figure S2 and Table2. The presence of hydroxyl groups and hydrated water molecules in CrL1 and MnL2 is responsible for the broadband that was visible in the complexes of CrL1, FeL1, CrL2, and MnL2’s spectra between 3444 and 3389 cm^−1^. Additionally, the coordinated water's rocking and wagging modes may be responsible for the bands that first formed at 976–961 cm^−1^. The existence of an azomethine group (C = N) was suggested by two signals in the IR spectra of the HL1 and HL2 ligands at (1612 and 1610) cm^−1^ ^32^. The complexes’ spectra showed that these signals were shifted to the region between 1625 and 1594 cm^−1^, demonstrating coordination of the azomethine group with metal ions. The bidentate ligands also displayed a band at 1271 and 1290 cm^−1^ for the v(C–O) stretching vibration.The redshift of this band to 1265–1247 cm^−1^ during complex formation is proof that the oxygen atom in phenol was coordinated. Additional evidence for this may be seen in the presence of two bands at 580–536 and 475–452 cm^−1^, respectively, which are related to the stretching vibrations of the M–O and M–N bonds in the complexes^33^.

**Table 2:** IR spectral bands of the new Cr(III), Mn(II), and Fe(III) complexes and their ligands at 4000–400 cm^−1^.

Compounds	*v*(OH)/H_2_O	*V* (C = N)	*v*(C–O)	*v*( H_2_O) )_Coor_	*v*(M–O)	*v*(M–N)
HL1	3378	1612	1271	–		–
HL2	3379	1610	1290	–	–	–
CrL1	3444	1625	1250	975	580	475
FeL1	3389	1604	1250	961	565	452
MnL2	3442	1606	1265	976	536	470
CrL2	3436	1594	1247	973	539	459

### Electronic spectra

**Figure 2:**
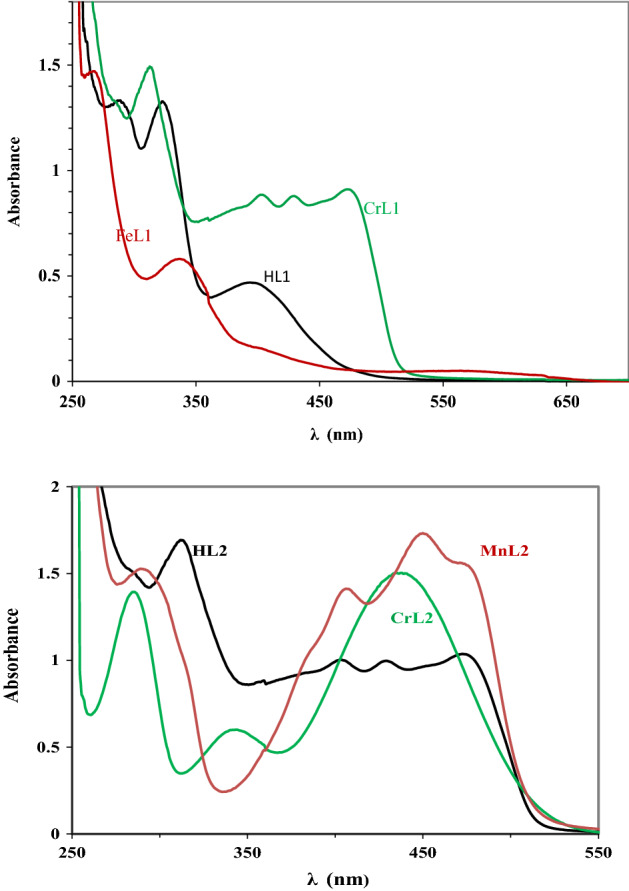
UV–vis spectra of the two ligands and their complexes with the metals CrL1, FeL1, CrL2, and MnL2 at concentrations of 10^−3^ M in DMF at 298.

At room temperature, DMF was used to record the electronic spectra of the ligands and their complexes with Cr(III), Mn(II), and Fe(III). These results are displayed in Fig. 2 and Table 3 respectively. Two distinct bands at max = 286 nm and 331, 327 nm in the spectra of HL1 and HL2 are attributable to π → π* and n → π* transition arising from the azomethine group. For HL1 and HL2, the intraligand band was seen at max = 387 and 381 nm, respectively.

**Table 3:** UV–vis spectra values of two ligands and their new FeL1, CrL1, CrL2 and MnL2 metal complexes.

Compounds	λ_max_ (nm)	Assignment	μ_eff_ (B.M.)
CrL1	286 398 439	^4^A_2g_(F) → ^4^T_2g_(F) ^4^A_2g_(F) → ^4^T_1g_(F) ^4^A_2g_(F) → ^4^T_1g_ (P)	4.01
FeL1	286 346 414	^6^A_1g_(S) → ^4^T_1g_(G) ^6^A_1g_(S) → ^4^T_2g_(G) ^6^A_1g_(S) → ^4^E_g_,^4^A_1g_(G)	5.19
CrL2	286 387 431	^4^A_2g_(F) → ^4^T_2g_(F) ^4^A_2g_(F) → ^4^T_1g_(F) ^4^A_2g_(F) → ^4^T_1g_ (P)	4.05
MnL2	288 408 452 477	^6^A_1g_ → ^4^T_1g_(^4^G) ^6^A_1g_ → ^4^E_g_(^4^G) ^6^A_1g_ → ^4^E_g_(^4^D) ^6^A_1g_ → ^4^T_1g_(^4^P)	5.22

In Table 3, lists of the metal complexes' electronic spectra and magnetic moments are provided. Three transitions are seen in the reflectance spectra of CrL1 and CrL2 at wavelengths of 475, 431, 406 and 315 nm and 435, 348 and 288 nm, respectively, which are brought about by the transitions ^4^A_2g_(F) → ^4^T_2g_(F), ^4^A_2g_(F) → ^4^T_1g_(F) and ^4^A_2g_(F) → ^4^T_1g_ (P), respectively which implies an octahedral geometry. The magnetic moment value was reported to be between 4.01 and 4.05 B. M., which is in good accord with the values for the Cr(III) complex in octahedral geometry that are already reported^28,31,34^. The transitions ^6^A_1g_ → ^4^T_1g_(^4^G), ^6^A_1g_ → ^4^E_g_(^4^G), ^6^A_1g_ → ^4^E_g_(^4^D) and ^6^A_1g_ → ^4^T_1g_(^4^P), in the solid reflectance spectra of the MnL2 complex at 477, 452, 408, and 288 nm^31^, respectively, can be due to the octahedral structure surrounding the Mn(II). The octahedral structure is supported by the measured value of 5.22 B. M. for the Mn(II) complex. The transitions are linked to three bands at 570, 409, and 337 nm that are seen in the Fe(III) complex's electronic spectra. Specifically, ^6^A_1g_(S) → ^4^T_1g_(G), ^6^A_1g_(S) → ^4^T_2g_(G) and ^6^A_1g_(S) → ^4^E_g_,. This compound has an octahedral structure with a high spin number^36^.

### Mass spectra

The mass spectra of all compounds displayed a variety of fragmentation patterns as was expected, and the results were found to be in good agreement with the molecular formulae of the compounds. According to the mass spectra of HL1, which displayed a molecular ion peak at m/z = 320.21 and HL2 at 271.93 a. m. u. (320.19 and 271.32 a. m. u.) in Figure S3, the proposed formulas C_15_H_14_NO_2_Br and C_16_H_17_NO_3_ are in a favorable range compared to the predicted value. The ESI–MS spectra of the CrL1, FeL1, CrL2 and MnL2 complexes after complexation are depicted in Figure S4. These spectra revealed molecular ion peaks at m/z = 496.42, 482.21, 428.23, and 433.12 a. m. u., respectively, which were coextensive with the calculated weights of 496.12, 481.95, 429.23, and 432.76 a. m. u. The complexes exhibited the molecular ion peak at m/z = 320.31, 321.34, 270.04 and 272.01 a. m. u. which corresponds to the parent ligands HL1 and HL2 confirms the formation of complexes.

### Thermal studies (TGA)

The use of TGA is essential for assessing the properties of new metal complexes, distinguishing the different solvent molecules that are present within or outside the coordination sphere, and figuring out the thermal stability of the complexes. In addition to the outcomes of the microanalyses, the results of the TGA, which was carried out between 20 and 800 °C, were utilized to assess and compute mass loss.

**Figure 3:**
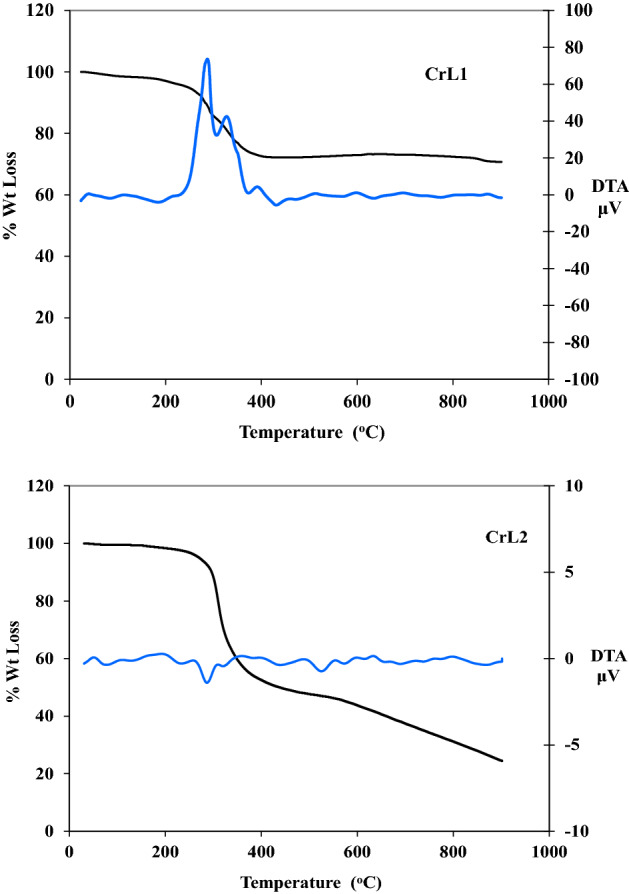
Results of a thermal study done on CrL1, and CrL2 complexes heated in air at a rate of 10 °C/min to 800 °C from room temperature to that temperature.

Table 4, Figs. (3 and S5) The TG curves of two ligands and the chelates CrL1, FeL1, CrL2 and MnL2 (4 and S4). At temperatures between 114 and 294 °C (Calc. 97.56 percent, found 97.43 percent) for HL1 and 155 and 304 °C (Calc. 99.33%, found 99.65%) for HL2, the ligands’ TGA plot shows their complete thermal breakdown in one step.

**Table 4:** Thermal analysis findings for CrL1, FeL1, CrL2, and MnL2 complexes heated at a rate of 10 °C/min to 800 °C from room temperature to 800 °C.

Complex	Temp. /°C DTG	Temp. range /°C TGA	Calc. Wt. loss % (F.)	Assignments
**CrL1** [C_15_H_17_BrCl_2_NO_4_Cr]H_2_O Residue	281 318 378	42–287 287–400 > 400	29.02 (28.96) 57.58 (57.54) 13.70 (13.67)	H_2_O_hyd._, 2H_2_O_coor._, 2HCl, OH C_15_H_12_BrN CrO
**FeL1** C_15_H_17_BrCl_2_NO_4_Fe Residue	61 310 450	182–303 303–680 > 680	31.95 (31.98) 53.55 (53.51) 14.92 (14.89)	2H_2_O_coor._, 2HCl, C_2_H_5_O C_13_H_8_BrN FeO
**CrL2** C_16_H_20_O_5_NCl_2_Cr Residue	278 431 531	178–299 299–398 398–582 > 582	8.39 (8.35) 68.54 (68.57) 7.22 (7.17) 15.84 (15.87)	2H_2_O_coor_ C_15_H_13_ONCl_2_ CH_3_O CrO
MnL2 [C_16_H_22_O_6_NClMn]H_2_O Residue	92 298 581	89–146 146–223 223–587 > 544	4.16 (4.23) 20.91 (20.89) 58.53 (58.51) 16.39 (16.34)	H_2_O_hyd_ 3H_2_O_coor._, HCl C_16_H_15_NO_2_ MnO

In the TG curves of the CrL1 and FeL1 complexes, three weight losses events can be seen. Weight losses of 28.96 and 31.98% (estimated at 29.02 and 31.95) are believed to indicate losses of two coordinated H_2_O molecules during the first breakdown stage, which took place between 42 and 303 °C + 2HCl, as well as one hydrated water molecule + hydroxyl group in the case of CrL1, and a loss of C_2_H_5_O in the FeL1 complex.

The second phase took place between 287 °C and 680 °C and involved the losses of the C_15_H_12_BrN and C_13_H_8_BrN moieties, with weight losses from the complexes estimated to be 57.54 and 53.51% (calculated as 57.58 and 53.55), respectively. With weight losses of 13.67 and 14.89% (calc. 13.70 and 14.92%) and DTG curve correlations at (281, 318, and 378 °C) and (62, 310, and 450 °C), respectively, CrO and FeO are the remaining final residues.

The initial degradation phase occurred between 89 and 299 °C and was followed by weight losses of 8.35 and 4.23% (calc. 8.39 and 4.16), which for the CrL2 and MnL2 complexes, respectively, amounted to the loss of two coordinated and one hydrated water molecule.

The TG curves for the CrL2 and MnL2 complexes show four weight loss events. The second process involved the loss of the C_15_H_13_ONCl_2_, three coordinated water molecules, and HCl moieties and occurred between 146 °C and 398 °C. In the third stage, which occurred between 22 °C and 587 °C, the complexes lost CH_3_O and C_16_H_15_NO_2_, with estimated weight losses of 7.17 and 58.51% (calculated as 7.22 and 58.53), respectively. As the final residues, CrO and MnO had weight losses of 15.78 and 16.34% (calculated weight losses of 15.84 and 16.39%), which are in agreement with the DTG curves at (281 °C, 318 °C and 378 °C) and (62, 310 and 450 °C), respectively.

### X-ray diffraction

**Figure 4:**
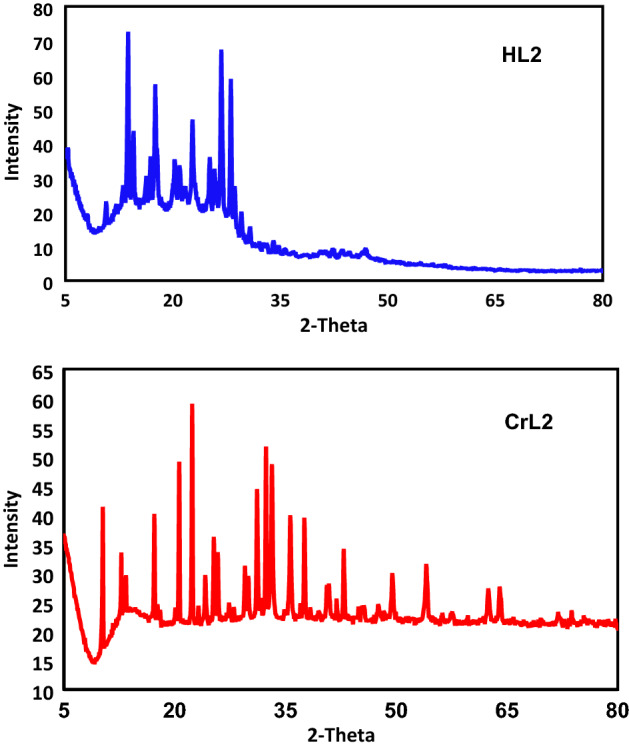
HL2 and its CrL2 complex’s PXRD pattern (**A**) (**B**).

Due to difficulties in obtaining single crystals of the produced metal complexes, Fig. 4 and Table 5 demonstrate the results of powder X-ray diffractions (PXRD) of the HL2 and its CrL2 complex over the 2θ range 5–80° using a wavelength of 1.5406 nm.

**Table 5:** Crystal system, d spacing values and crystal size of ligand HL2 and its CrL2 complex.

Compounds	Crystal system	2θ(Obs)	d(Obs)Å	Crystal size (nm)
HL2	Monoclinic P1211(4)	26.757	3.325	33.70
CrL2	Cubic F m − 3 m (225)	22.374	3.970	60.27

The lattice constants of the HL2 ligand’s unit cell, which changed into noteworthy, are as follows: a = 8.322, b = 6.8010, c = 12.8850, = 91.74°, and unit cell volume V = 728.93 Å^3^.

At least 10 distinct, highly intense diffraction peaks were visible in the ligand HL2’s diffractogram at 2θ = 13.74°, 14.32°, 15.22°, 18.62°, 21.23°, 23.52°, 26.48°, 27.75°, 28.40°, 30.13° with maxima at 2θ = 13.74°, which corresponds to an inter-planar spacing (d) value of 6.44 Å. As for the CrL2, the acquired unit mobile yielded values of lattice constants, a = 12.3488 Å and unit cell volume of V = 1883.10 Å^3^. These cellular parameters performed the situation wherein a = b = c and α = β = γ = 90° as required for the pattern to be cubic.

Thus, it can be concluded that Cr(III) complex has only one purely crystalline phase having a monoclinic crystal system. More than fifteen strong diffraction peaks may be found in the XRD pattern of the CrL2; the feature ones are regarded as 2θ = 12.05°, 20.32°, 23.88°, 24.96°, 28.90°, 31.56°, 32.40°, 37.83°, 41.33°, 43.96°, 46.47, 52.91 with maxima at 2θ = 22.38° corresponding to d value of 3.969 Å. The average particle size (dXRD) in Table 5 turned into calculated the use of Scherer’s equation^32,33^. Where t = 0.9 /B cos θ, t = 0.9 /B cos, where t is the crystal thickness (in nm), B is the half-width (in radians), is the Bragg angle, and is the wavelength, is the formula for calculating the particle size. The particle size corresponding to each diffraction maximum is calculated from the half-width of the diffraction peak. CrL2 complex crystals measure 60.27 nm on average.

### DFT study

As single crystals of the ligand and complexes could not be received notwithstanding the first-rate efforts, molecular modeling has been employed to higher understand the geometrical structures of the compounds presently studied.

#### Molecular DFT calculation of two ligands:

**Figure 5:**
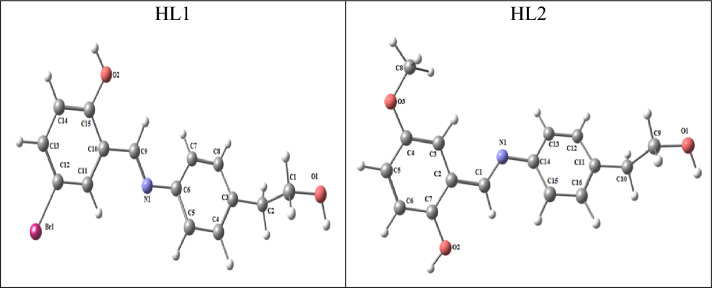
The natural charges on active centers as well as the HL1and HL2 optimal structures.

Figure 5 shows the optimized forms of the ligands HL1 and HL2 as the lowest energy structures. O_1_, O_2_, and N_1_ are the more detrimental active sites for L_1_, whereas O_1_, O_2_, O_3_, and N_1_ are the more detrimental active sites for HL2. These natural charges were discovered via Natural Bond Orbital Analysis (− 0.506). (NBO).

So, rather of O_2_ and N_1_ creating a stable 6-membered ring for both, the metal ions choose the bidentate coordination.

#### Molecular DFT calculation of [Fe(L_1_)(H_2_O)_2_***Cl***_2_]:

**Figure 6:**
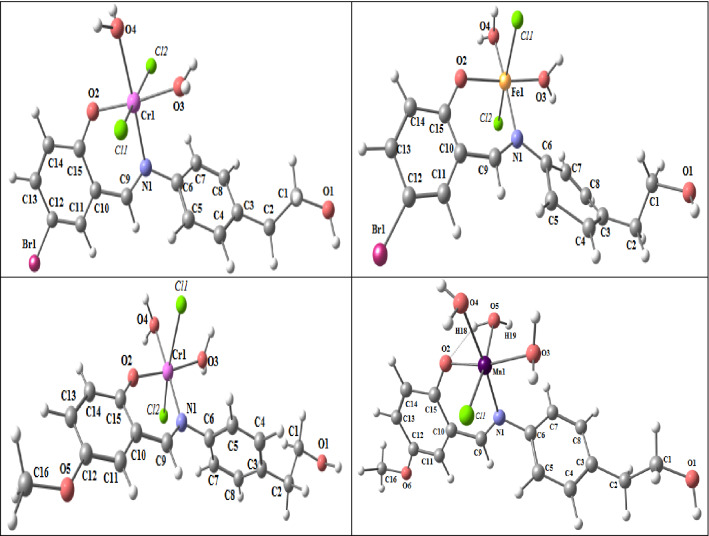
The optimized structures of [Fe(L1)(H_2_O)_2_*Cl*_2_] (**a**), [Cr(L1)(H_2_O)_2_*Cl*_2_], up, and [Cr(L2)(H_2_O)_2_*Cl*_2_] down (**b**) and of [Mn(L2)(H_2_O)_3_*Cl*] (**c**).

Figure 6 depicts the complex [Fe(L1)(H_2_O)_2_*Cl*_2_] optimized ]'s structure in its lowest energy configuration.

The six-coordinated octahedral structure of the iron atom has the elements N_1_, O_2_, O_4_, and O_3_ virtually on a single plane with a 0.402° deviation (Table 6). As a result of N_1_ and O_2_ coordinating, there is a space between N_1_ and O_2_ decreases from 4.107 (in free L_1_) to 2.789 (in the complex [[Fe(L1)(H_2_O)_2_*Cl*_2_]). The coordinated atoms in [Fe(L1)(H_2_O)_2_*Cl*_2_]) have the following natural charges calculated from the NBO analysis: Fe (+ 0.405), O_2_(− 0.579), N_1_(− 0.433), O_3_(− 0.870), O_4_(− 0.870), Cl_1_(− 0.387), and Cl_2_ (− 0.391).

**Table 6:** The optimized bond lengths (Å) and bond angles (°) of [Fe(L1)(H_2_O)_2_*Cl*_2_].

Type of bond	Bond length(Å) Fe(L1)	Type of bond	Bond length(Å) Fe(L1)
Fe–N_1_	1.977	Fe–O_4_	1.865
Fe–O_2_	2.010	Fe–*Cl*_1_	2.078
Fe–O_3_	1.852	Fe–*Cl*_2_	2. 414
Type of bond	Angle (°) Fe(L1)	Type of bond	Angle (°) Fe(L1)
N_1_–Fe–O_2_ N_1_–Fe–O_3_ O_2_–Fe–O_4_ O_3_–Fe–O_4_ *Cl*_1_–Fe–N_1_ *Cl*_1_–Fe–O_2_ *Cl*_1_–Fe–O_3_ *Cl*_1_–Fe–O_4_	94.26 83.83 84.90 99.15 100.1 91.79 84.31 86.62	*Cl*_2_–Fe–N_1_ *Cl*_2_–Fe–O_2_ *Cl*_2_–Fe–O_3_ *Cl*_2_–Fe–O_4_ N_1_–Fe–O_4_ O_2_–Fe–O_3_ *Cl*_1_–Fe–*Cl*_2_ N_1_–O_2_–O_4_–O_3_	89.88 95.27 93.84 85.10 178.1 172.3 165.4 1.402*

^•^dihedral angle.

#### Molecular DFT calculation of [Cr(L1)(H_2_O)_2_***Cl***_2_] and [Cr(L_2_)(H_2_O)_2_***Cl***_2_]:

Figures 6 exhibits the lowest energy configurations of the complexes [Cr(L1)(H_2_O)_2_*Cl*_2_] and [Cr(L_2_)(H_2_O)_2_*Cl*_2_] in their optimized structures. The chromium atom is organized in an octahedron with six coordinated bonds, and in the complexes [Cr(L1)(H_2_O)_2_*Cl*_2_] and [Cr(L_2_)(H_2_O)_2_*Cl*_2_], the atoms N_1_, O_2_, O_4_, and O_3_ are almost parallel to one another with deviations of 0.287° and 0.571°, respectively.

Table 7. The distance between N_1_ and O_2_ is decreased from 4.107 Å and 4.109 Å (in free L_1_ and L_2_) to 2.787 Å and 2.764 Å (in the complexes [Cr(L1)(H_2_O)_2_*Cl*_2_] and [Cr(L_2_)(H_2_O)_2_*Cl*_2_], respectively) due to coordination of both N_1_ and O_2_. The natural charges computed from the NBO analysis on the coordinated atoms in [Cr(L1)(H_2_O)_2_*Cl*_2_] are Cr (+ 0.402), O_2_(− 0.554), N_1_(− 0.443), O_3_(− 0.882), O_4_(− 0.879), *Cl*_1_(− 0.368) and *Cl*_2_(− 0.387). The natural charges computed from the NBO analysis on the coordinated atoms in [Cr(L2)(H_2_O)_2_*Cl*_2_] are: Cr (+ 0.385), O2(− 0539), N1(− 0.436), O3(-0.880), O4(− 0.877), *Cl*1(− 0.382) and *Cl*2(− 0.399).

**Table 7:** Bond angles (°) and important optimum bond lengths (Å) of [Cr(L1)(H_2_O)_2_*Cl*_2_] and [Cr(L2)(H_2_O)_2_*Cl*_2_].

Type of bond	Bond length (Å) Cr(L1)Cr(L2)	Type of bond	Bond length (Å) Cr(L1)Cr(L2)
Cr–N1	2.020 2.027	Cr–O_4_	2.075 2.084
Cr–O2	1.873 1.858	Cr–*Cl*_1_	2.379 2.381
Cr–O3	2.042 2.053	Cr–*Cl*_2_	2.375 2.394
Type of bond	Angle (°) Cr(L1)Cr(L2)	Type of bond	Angle (°) Cr(L1)Cr(L2)
N_1_–Cr– O_2_ N_1_–Cr–O_3_ O_2_–Cr–O_4_ O_3_–Cr–O_4_ *Cl*_1_–Cr–N_1_ *Cl*_1_–Cr–O_2_ *Cl*_1_–Cr–O_3_ *Cl*_1_–Cr–O_4_	91.24 90.61 93.86 94.97 86.16 87.27 88.73 87.14 96.77 92.66 97.07 96.06 84.07 82.44 84.32 87.15	*Cl*_2_–Cr–N_1_ *Cl*_2_–Cr–O_2_ *Cl*_2_–Cr–O_3_ *Cl*_2_–Cr–O_4_ N_1_–Cr–O_4_ O_2_–Cr–O_3_ *Cl*_1_–Cr–*Cl*_2_ N_1_–O_2_–O_4_–O_3_	96.77 97.26 97.07 97.19 82.44 83.39 84.07 83.43 177.3 177.8 174.6 174.3 163.7 163.3 0.623* 0.571*

*dihedral angle.

#### Molecular DFT calculation of [Mn(L2)(H_2_O)_3_***Cl***]:

Figure 6 revealed the lowest energy configuration structure of [Mn(L2)(H_2_O)_3_*Cl*] complex.

The coordination number of the Mn atom is six and the N_1_, O_2_, O_4_, and O_3_ atoms of the complex are almost in the same plane but are displaced by 10.60 degrees suggesting an octahedral geometry of the complex.

Due to the coordination of both N_1_ and O_2_, the distance between N_1_ and O_2_ is reduced from 4.107 and 4.109 (in free L2) to 2.838 (in the complex [Mn(L_2_(H_2_O)_3_*Cl*]) Table 8.

**Table 8:** Important optimized bond lengths (Å) and bond angles (°) of [Mn(L2)(H_2_O)_2_*Cl*_2_].

Type of bond	Bond length (Å) Mn(L2)	Type of bond	Bond length (Å) Mn(L2)
Mn–N_1_	1.980	Mn–O_4_	2.102 2.155 2.533
Mn–O_2_	1.944	Mn–O_5_	
Mn–O_3_	2.098	Mn–*Cl*_1_	
Type of bond	Angle (°) Mn(L2)	Type of bond	Angle (°) Mn(L2)
N_1_–Mn–O_2_ N_1_–Mn–O_3_ O_2_–Mn–O_4_ O_3_–Mn–O_4_ *Cl*_1_–Mn–N_1_ *Cl*_1_–Mn–O_2_ *Cl*_1_–Mn–O_3_ *Cl*_1_–Mn–O_4_	92.60 96.16 86.92 85.32 93.24 105.3 79.92 78.34	O_5_–Mn–N_1_ O_5_–Mn–O_2_ O_5_–Mn–O_3_ O_5_–Mn–O_4_ N_1_–Mn–O_4_ O_2_–Mn–O_3_ *Cl*_1_–Mn–O_5_ N_1_–O_2_–O_4_–O_3_	101.2 76.59 96.03 87.32 171.1 169.5 165.3 10.24*

*dihedral angle.

The coordinated atoms in [Mn(L2)(H_2_O)_3_*Cl*] have natural charges of Mn (+ 0.581), O_2_(− 0.745), N_1_(− 0.490), O_3_(− 0.904), O_4_(− 0.921), O_5_(− 0.904), and Cl_1_ (− 0.564).

**Figure 7:**
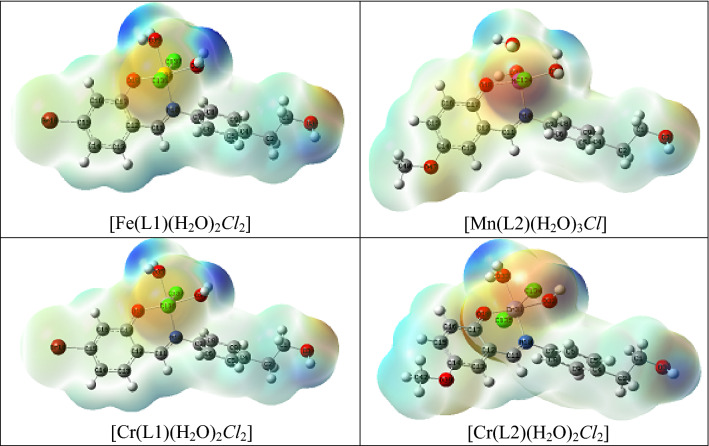
Molecular electrostatic potential (MEP) surface of ligands (HL1 and HL2) and their metal complexes.

**Figure 8:**
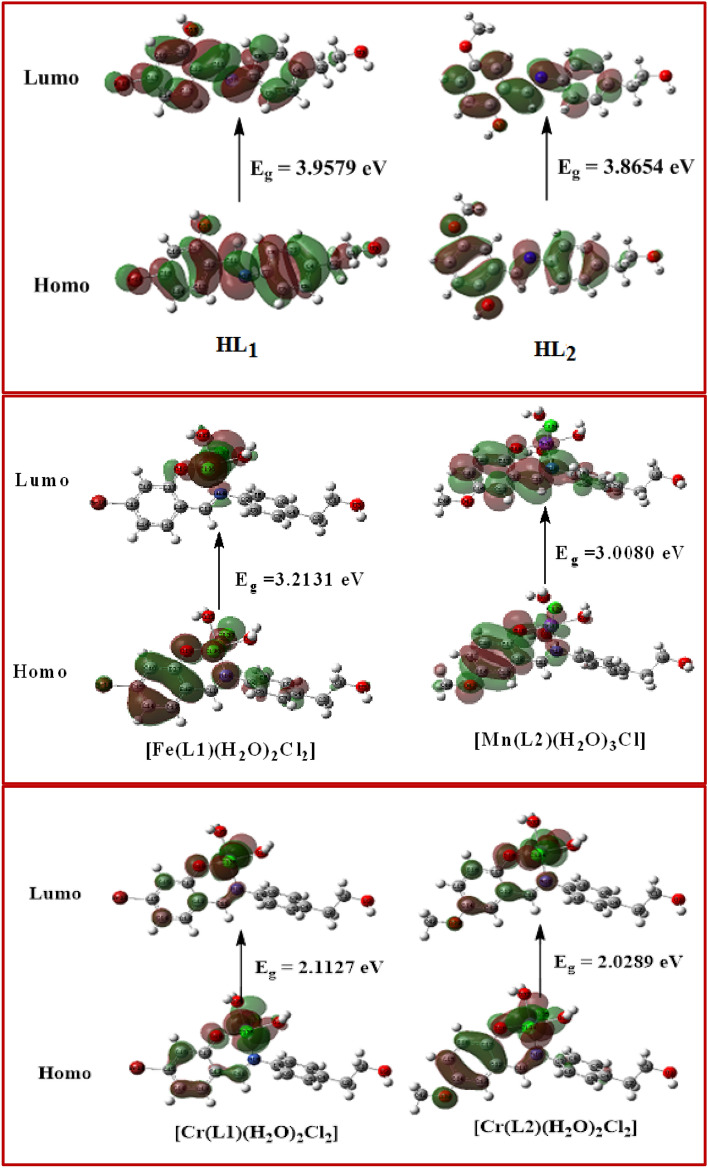
HOMO and LUMO charge density maps of two ligands and their metal complexes.

Figure 7 demonstrates how crucial the MEP surface is in identifying the positive (blue; loosely bound or with additional electrons) and negative (red; contains surplus electrons) charged electrostatic potentials of the molecule. The estimated HOMO (highest occupied molecular orbital energies), LUMO (lowest unoccupied molecular orbital energies), and total energy were all calculated for the ligands and complexes (Table 9). Energy gap (E_g_) = E_LUMO_ − E_HOMO_. The total energy of the complexes decreases as a result of the chelation of the ligand to metal ions, and this decrease in total energy suggests that the complexes are more stable than the free ligands. This is seen in Table 9. Because Eg in complexes is smaller than it is in the free ligand, the charge transfer interactions shown in Fig. 8 may be understood.

**Table 9:** Calculated energies of two ligands and their complexes.

Compounds	E^a^	HOMO^b^	LUMO^c^	E_g_^d^	Dipole moment^e^
HL1	− 798.25	− 6.0203	− 2.0624	3.9579	0.9339
HL2	− 900.20	− 5.7278	− 1.8624	3.8654	3.2991
[Fe(L1)(H_2_O)_2_*Cl*_2_]	− 1104.02	− 6.3503	− 3.1372	3.2131	3.3196
[Cr(L1)(H_2_O)_2_*Cl*_2_]	− 1066.89	− 5.6935	− 3.6932	2.0003	2.1023
[Cr(L_2_)(H_2_O)_2_*Cl*_2_]	− 1168.85	− 5.3302	− 3.3013	2.0289	3.2549
[Mn(L2)(H_2_O)_3_*Cl*]	− 1247.88	− 4.6611	− 1.6531	3.0080	4.2051

^a^*E* the total energy (a. u.). ^b^*HOMO* highest occupied molecular orbital (eV). ^c^*LUMO* lowest unoccupied molecular orbital (eV).

^d^E_g_ = E_LUMO_ − E_HOMO_ (eV). ^e^dipole moment (Debye).

### Biological study

#### Antimicrobial activity

**Figure 9:**
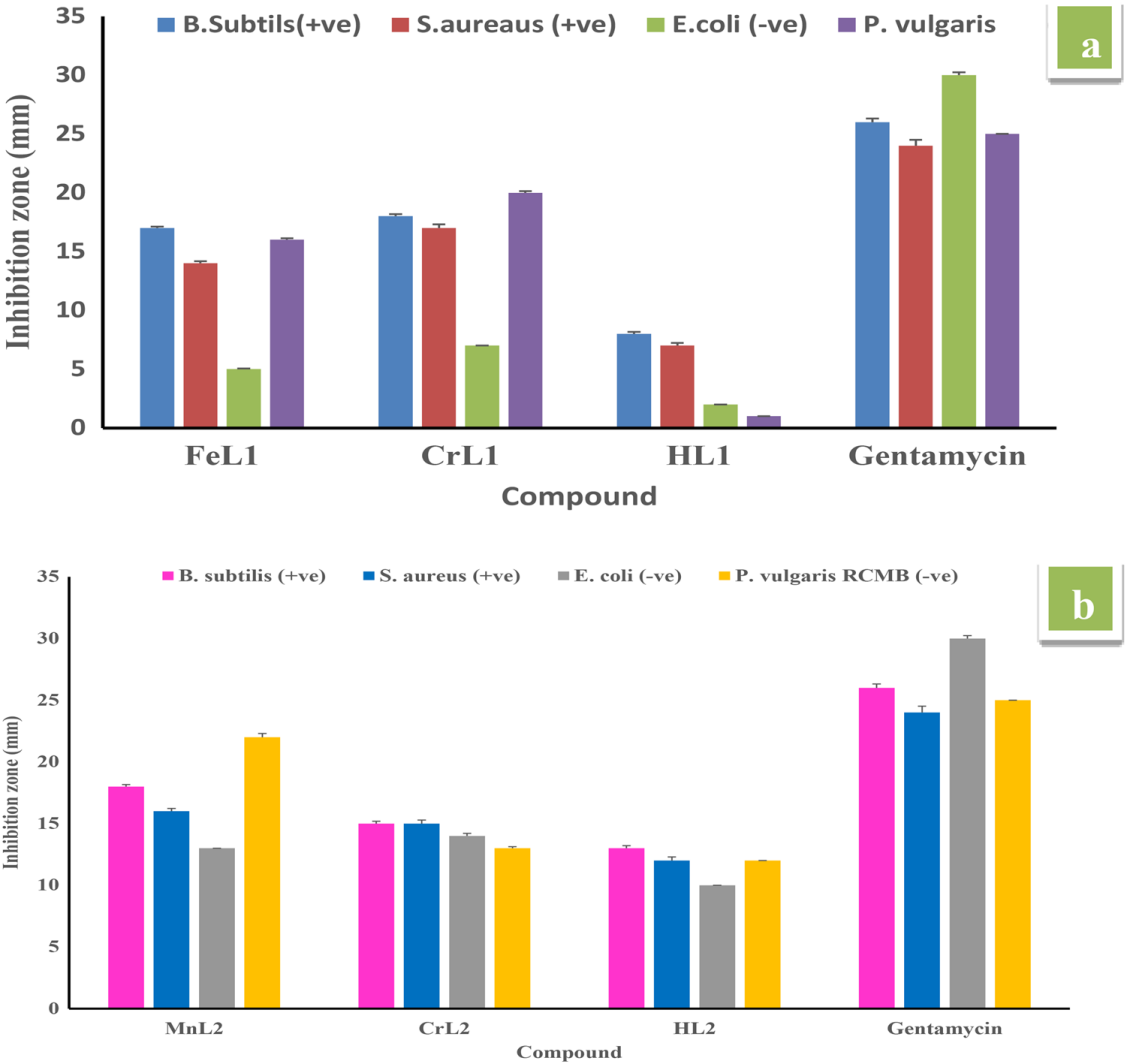
Inhibition zone and their standard deviation values of (**a**) of HL1 ligand and its FeL1 and CrL1 complexes and (**b**) of HL2 ligand and its MnL2 and CrL2 complexes against *S. aureus(*+ *ve), B. subtilis (*+ *ve), P. vulgaris (− ve) and E. coli (− ve)* at 20 μg/ml.

**Figure 10:**
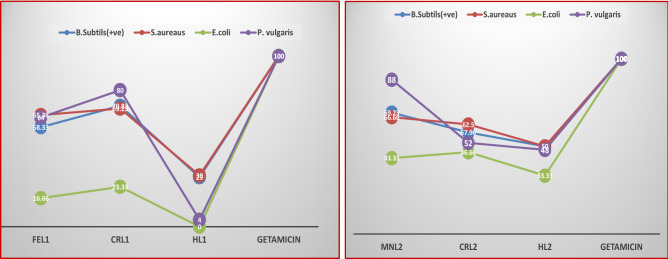
Activity index % values of HL1, HL2 and their metal complexes.

The effectiveness of the ligands (HL1 and HL2) and their Cr(III), Fe(III), and Mn(II) metal complexes to inhibit the growth of the bacterial species *Bacillus RCMB 015(1) NRRLB-543, Staphylococci aureus ATCC25923, Pseudomonas vulgaris RCMB 004(1) ATCC 13,315, Escherichia coli ATCC 25,922, and fungal species (RCMB 002,008).* The results presented in Tables S1, S2, and Fig. 9 demonstrate that the newly synthesized Cr(III), Fe(III), and Mn(II) complexes outperformed Schiff base HL1 in terms of antibacterial activity and followed the sequence CrL1 > FeL1 > HL1 when compared to gentamycin and ketoconazole. Moreover, complex MnL2 shows greater antimicrobial activity (inhibition zone) towards all the bacterial strains compared to CrL2 complex and HL2 (Table S1) and follows the order MnL2 > CrL2 > HL2. According to Tweedy’s chelation theory, the existence of chelation between the coordination sites of ligands (N, O) and Cr(III), Fe(III), and Mn(II) ions accounts for the higher antibacterial activity of metal complexes relative to free ligands^26,34^. Compared to the ligand HL1, which has an electron-withdrawing bromo group, the antibacterial activity of ligand HL2 which contains an electron-donating methoxy group in the salicylaldimine moiety has a powerful antibacterial and antifungal effectiveness Fig. 10 and Table S2. The methoxy group of the aldehyde moiety of the Schiff base ligand may help to explain why the vanillin-based ligand HL2 has a greater activity than the 5-bromo salicylaldehyde-based ligand. It was possible to connect the Schiff base activity to the electronic effects of the substituent since the bromo-analogue was less active than the methoxy-analog. When compared to free ligands, metal complexes have a higher antibacterial activity because chelation is predicted to improve the lipophilicity of the metal ion. MnL2 demonstrated stronger activity on chelation than CrL2 and HL2 ligand.

Thus, a crucial component of our research for the future is to comprehend how possible antimicrobial drugs work. Increased lipophilicity, a significant element determining bacteriostatic action, might be referred to as the HL2 ligand's stronger action than the HL1 ligand. Structure–activity correlations for the salicylidene fragment substitution show that the methoxy group, a donor group of the benzene ring, enhances the electron density over the azomethine group. The higher lipophilicity may be due to -electron delocalization over the entire chelating system. However, adding a bromo group, which is the benzene ring's withdrawing group, decreases the electron density over the azomethine group and the entire chelating system, potentially lowering HL1’s lipophilicity in comparison to HL2 and, as a result, its antimicrobial activity.

#### The mechanism of action for metal complexes used as antibacterial agents

Tweedy’s chelation theory states that the coordination of metal ions to ligands causes a reduction in the polarity of the metal atoms by delocalizing -electrons over the whole chelate and transferring some positive charge with donor groups. The chelates' ability to penetrate the lipid membrane of the bacterial cell is improved by increasing their lipophilicity as a result. The various properties of the metal complexes may increase their activity after chelation. By sharing a portion of its positive charge with donor groups and overlapping the orbitals of its ligands, the metal ion's polarity is diminished^39^. This process makes it easier for the complex to enter the lipid membrane of the bacteria and create an unbreakable covalent bond that obstructs the metal binding sites in their enzymes^40^.

#### In vitro anticancer activity

**Figure 11:**
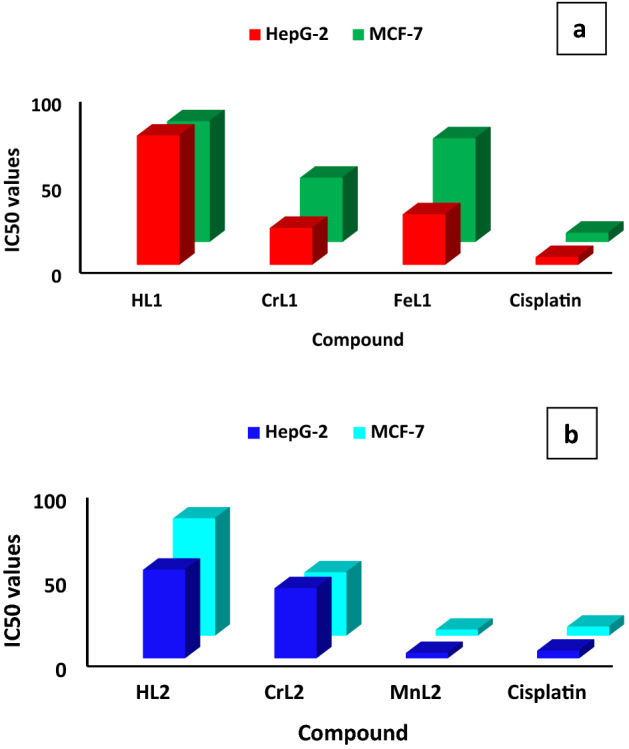
Cytotoxicity of HL1 and its CrL1 and FeL1 complexes (**a**) and HL2 and its CrL2 and MnL2 complexes (**b**) against MCF-7 and Hep-G2 compared to cisplatin.

The intriguing outcomes of the molecular docking of the two Schiff bases, HL1 and HL2, and their Cr(III), Fe(III), and Mn(II) complexes motivated the evaluation of the cytotoxic potential against the Hep-G2 hepatocellular carcinoma cancer cell line and the MCF-7 human breast cancer cell line. To assess the two ligands' anticancer properties, together with those of their Mn(II), Fe(III), and Cr(III) complexes, cisplatin was utilised as a positive control. Table S3 and Fig. 11 display the results. All the new complexes showed significant activity, the IC_50_ values show that the effectiveness of the HL1 and its Fe(III) and Cr(III) complexes follow the order CrL1(IC_50_ 37.0, 29.0 μg/ml) < FeL1 (IC_50_ 60.0, 21.0 μg/ml) < HL1 (IC_50_ 70.0, 75.0 μg/ml) for the cell types Hep-G2 and MCF-7, respectively. Furthermore, the IC_50_ values of the ligand HL2 and its Mn(II) and Cr(III) metal complexes were in the following order for the MCF-7 and Hep-G2 cell lines: MnL2 (IC_50_ 3.0, 2.6 g/ml), CrL2 (IC_50_ 37.0, 41.0 g/ml), and HL2 (IC_50_ 69.0, 52.0 g/ml). Surprisingly, MnL2 had a higher cytotoxic potency than the reference medication cisplatin under the same conditions where the IC_50_ value of the complex MnL2 against MCF-7 and Hep-G2 (IC_50_ = 3.0, 2.6 μg/ml) is less than the standard drug cisplatin (IC_50_ = 4.8, 4.0 μg/ml), respectively. This finding has revealed that the MnL2 complex might be a promising therapeutic agent (Fig. 11). The strong propensity to bind DNA via the groove and intercalation modes, as well as the possibility for the Mn(II) center to become more involved in the generation of reactive oxygen species (ROS), which may both be signs of the intense activity include the ability to interfere with the transcription of cancer cell DNA and to trigger cell death through apoptosis. The complexes' capacity to bind DNA and their cytotoxicity are well-aligned.

#### DNA binding investigations

##### Studies of electronic spectral data

**Figure 12:**
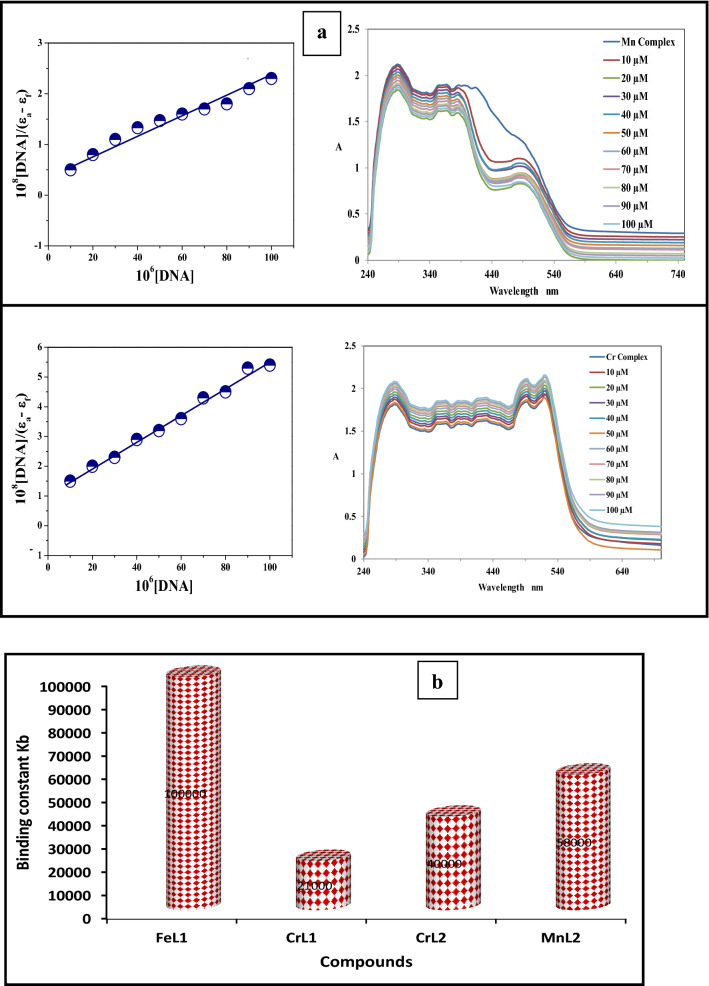
(**a**) MnL2 and CrL2 electronic spectra in Tris buffer, both with and without CT-DNA (pH 7.5, 298 K), the concentration of complex was 1 × 10^−3^ M^−1^ and CT-DNA concentrations were 10–100 μM corresponding to the curves from 1 to 10, respectively. (**b**) FeL1, CrL1, CrL2, and MnL2’s binding constant Kb in mol^−1^ dm^3^ values.

Examining the binding mode of a single molecule to DNA may be done with great use, distinction, and dependability using the absorption titration approach^35^. The change in compound structure has a significant impact on the absorption maxima, which is very sensitive to the absorption technique. We have observed the absorption maxima with a steady addition of DNA to ascertain the binding affinity of the novel compounds to CT-DNA. If there is hypochromism, either with or without a redshift, intercalation of non-covalent interaction may occur. Hyperchromism, on the other hand, could be a sign of electrostatic binding. Metal complexes at a constant concentration (10^−3^ M) were titrated with increasing concentrations of CT-DNA between 10 and 100 µM to conduct an electronic spectrum titration. The electronic spectra of the metal complexes MnL2, CrL2, CrL1, and FeL1 in the presence and absence of CT-DNA are shown in Figure 12a and Figure S6. The lowest energy bands are observed at 403 nanometers and are attributed to metal-to-ligand charge transfer (MLCT) transitions. Sharp bands at about 246 nm might be the result of intra-ligand π-π^*^ transitions.

The observed absorption spectra showed that the addition of DNA to the FeL1 and MnL2 complexes causes a significant hypochromism and a little blue shift at the MLCT band, which may reflect the intercalative binding mechanism of chemicals to CT-DNA. However, after titrating with CT-DNA, CrL1 and CrL2 displayed hyperchromic shift. The variations in absorbance in the MLCT band of the FeL1, CrL1, MnL2, and CrL2 complexes as CT-DNA concentration rose were tracked using the functional equation below to calculate the intrinsic binding constants (Kb) of the complexes^36–38^:

3 $$\frac{{\left[ {DNA} \right]}}{{\left( {\varepsilon_{a} - \varepsilon_{f} } \right)}} = \frac{{\left[ {DNA} \right]}}{{\left( {\varepsilon_{b} - \varepsilon_{f} } \right)}} + \frac{1}{{\left[ {K{}_{b}\left( {\varepsilon_{b} - \varepsilon_{f} } \right)} \right]}}$$

The calculated *K*_*b*_ values for the new complexes in Fig. 12b and Table 10 are in the following order: **FeL1** > **MnL2** > **CrL2** > **CrL1** complex. The negative free energy values of the new complexes indicate that the complex–DNA interaction is a spontaneous process.

$${\text{Chromism }}\left( {\text{\% }} \right)^{a} = {\raise0.7ex\hbox{${Abs _{free} - Abs_{bound} }$} \!\mathord{\left/ {\vphantom {{Abs _{free} - Abs_{bound} } {Abs _{free} }}}\right.\kern-0pt} \!\lower0.7ex\hbox{${Abs _{free} }$}}$$

**Table 10:** Values for the spectral parameters that govern how FeL1, CrL1, MnL2, and CrL2 complexes interact with CT-DNA.

Complex	λ _max_ free(nm)	λ _max_ bound (nm)	∆n	Chromism (%)^a^	Type of chromism	Binding constant $\times$ 10^4^	∆G kJ mol^−1^
FeL1	291 355 544	289 354 545	2 1 1	2 2 34	Hypo	10.0	− 28.5
CrL1	288 334 363 451 488	287 328 353 450 477	1 6 10 1 11	13 21 8 14 1	Hyper	2.1	− 24.7
MnL2	288 370 387 417	282 371 386 432	6 1 1 14	13 3 1 9	Hypo	5.8	− 27.2
CrL2	288 334 367 387 430 450 492 522	286 333 368 386 432 452 490 520	2 1 1 1 2 2 2 2	4 5 5 4 4 4 4 0.5	Hyper	4.0	− 26.3

##### The suggested mode of DNA interaction

**Scheme 2:**
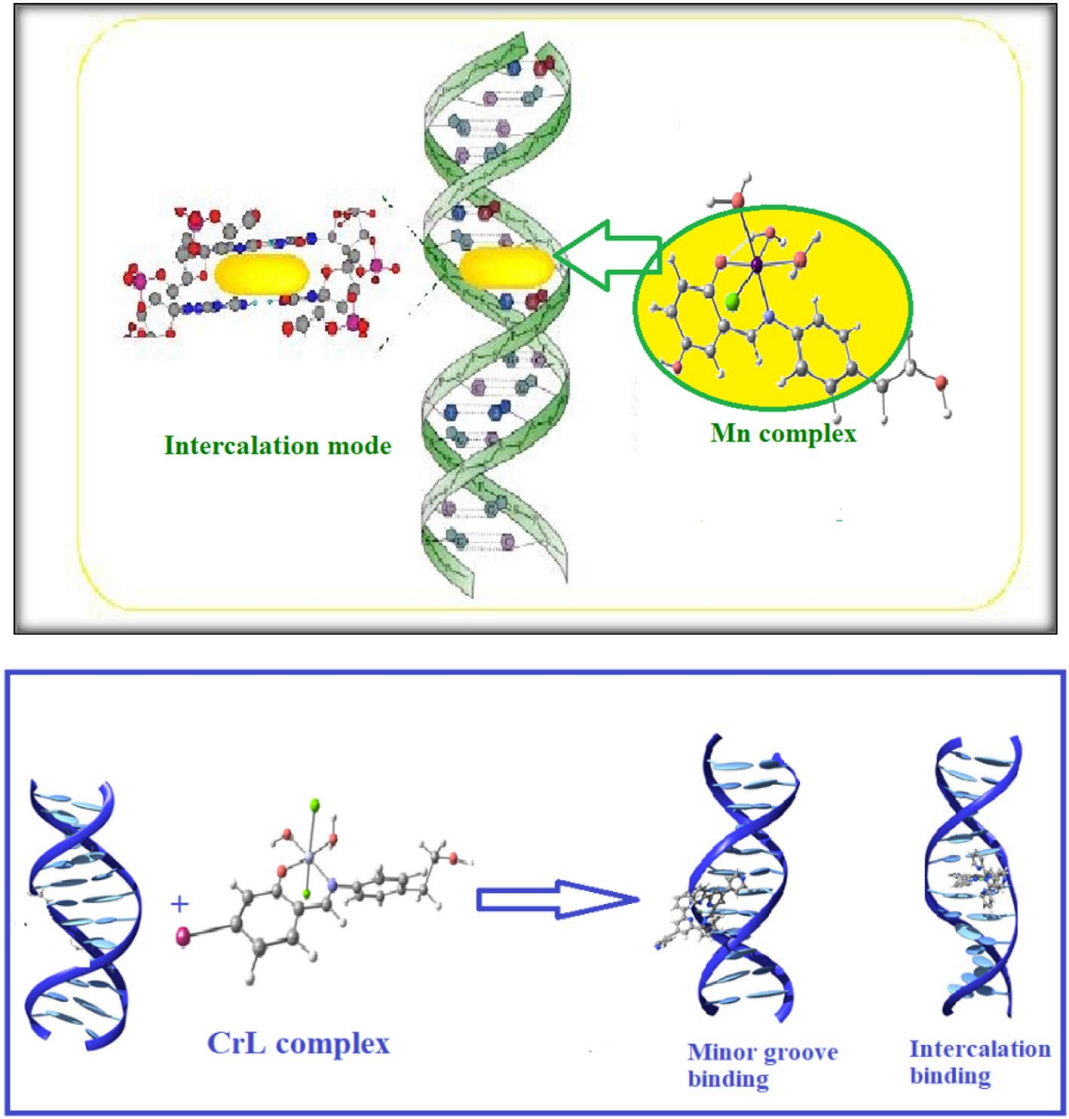
Mechanism of the interaction of MnL2 and CrL2 complexes with CT-DNA through intercalation of the complex in the base pairs stack of the DNA two helix.

The correlation of spectroscopic properties with hydrodynamic measurements between the novel complexes and DNA may reveal a variety of binding modalities. Most likely, the complex and DNA interact electrostatically or hydrophobically. The H_2_O molecules in the solution replace the chloride ligand in the MnL2 complex, giving it a positive charge^39–41^, this encourages electrostatic interaction between the MnL2 complex ion and the negatively charged phosphate group backbone at the outer border of CT-DNA double helix. As a result of the chloride ligand being removed from the complex in solution, the complexes will also have a flat zone in the center. Therefore, the MnL2 and CrL2 complexes may engage in the following DNA interactions: (Schemes 2). The initial stage of the CrL2 complex’s interaction mechanism is the electrostatic contact of the coordination sphere with the DNA base pairs or the interaction of the Cr(III) complex with the DNA base backbone. Additionally, when the complex’s flat component is positioned in between the DNA base pairs, Cr(III) interacts with the base pairs in a coordinated manner.

##### *DNA interaction *via* gel electrophoresis*

**Figure 13:**
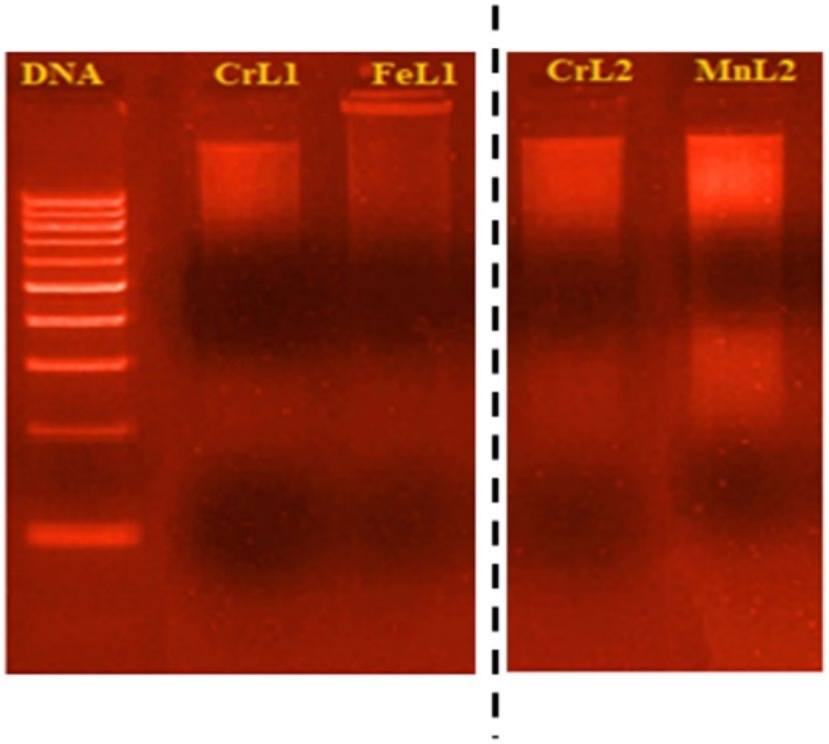
The cropped gel electrophoresis photograph demonstrating the new complexes' interaction with CT-DNA during binding. DNA ladder in lane 1, CrL1 and DNA in lane 2, FeL1 and DNA in lane 3, CrL2 and DNA in lane 4, and MnL2 and DNA in lane 5.

Gel electrophoresis is an extensively employed method to investigate the attachment of chemicals to nucleic acids. When subjected to an electric field, DNA flows toward the anode because it is negatively charged^47,48^. The intensity of the electric field, the buffer, the density of the agarose gel, and the size of the DNA all have an impact on how much DNA migrates. It has been established that DNA size and mobility are often negatively associated. The graphic shows the different bandwidths and brightness levels of the bands relative to the control. We investigated how the new complexes interacted with DNA using agar gel electrophoresis and the outcomes are displayed in Figure 13. (Lane 1 contains CT-DNA and a blank; Lanes 1 and 2 include CT-DNA and a metal complex.) The different DNA binding affinities of the novel complexes were assumed to be the cause of the variations in DNA-cleavage effectiveness. The growing lane intensity of **FeL1>MnL2>CrL2>CrL1** is in good agreement with the values of the CT-DNA binding constant. (Table 10).

#### Molecular docking study

**Figure 14:**
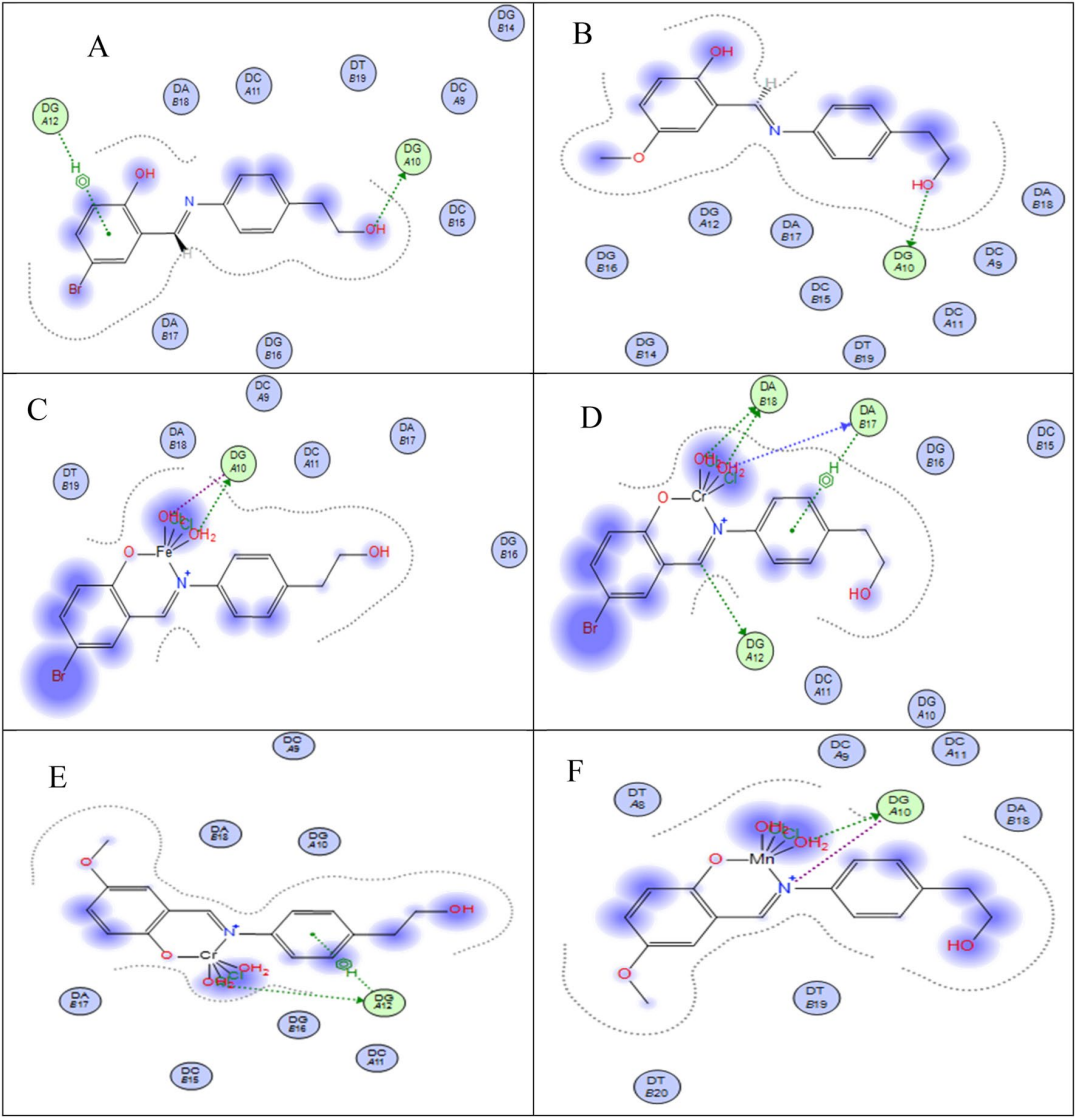
HL1 and HL2 interactions with the human DNA receptor site are shown in a 2D plot together with [Fe(L1)(H_2_O)_2_*Cl*_2_](C), [Cr(L1)(H_2_O)_2_*Cl*_2_](D), [Cr(L2)(H_2_O)_2_*Cl*_2_] (E) and [Mn(L2)(H_2_O)_3_*Cl*](F).

**Figure 15:**
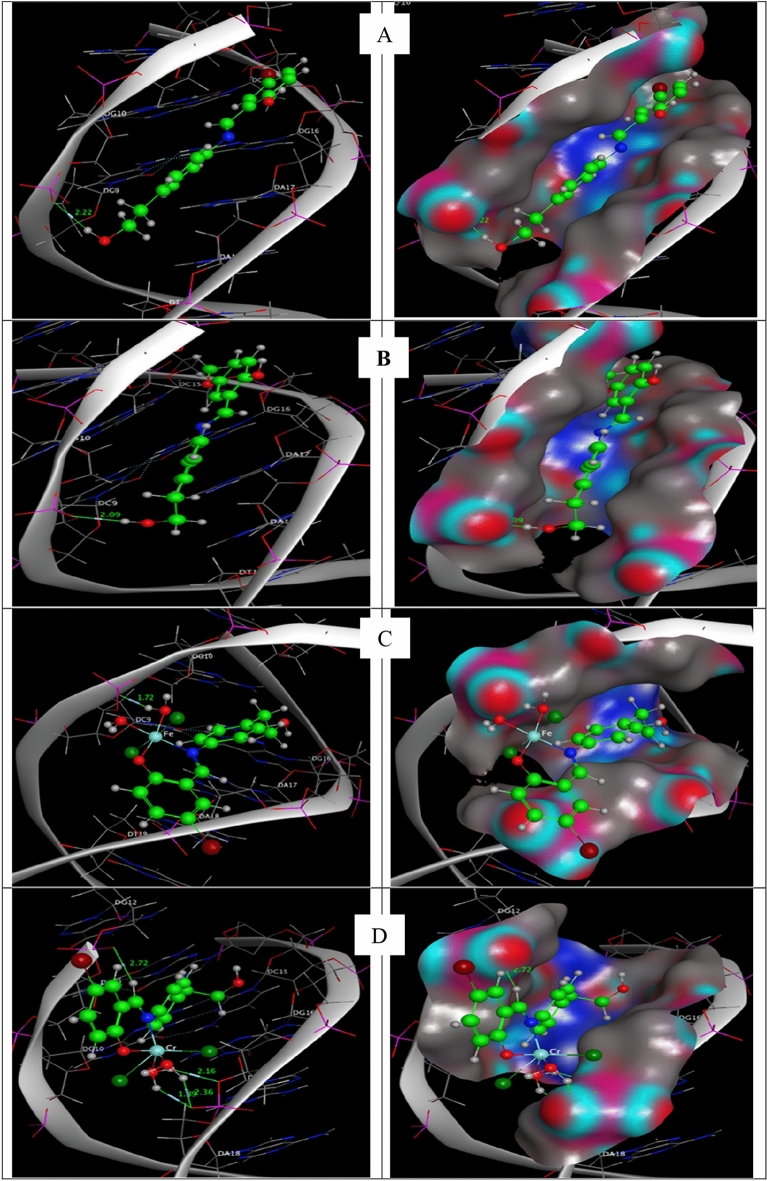

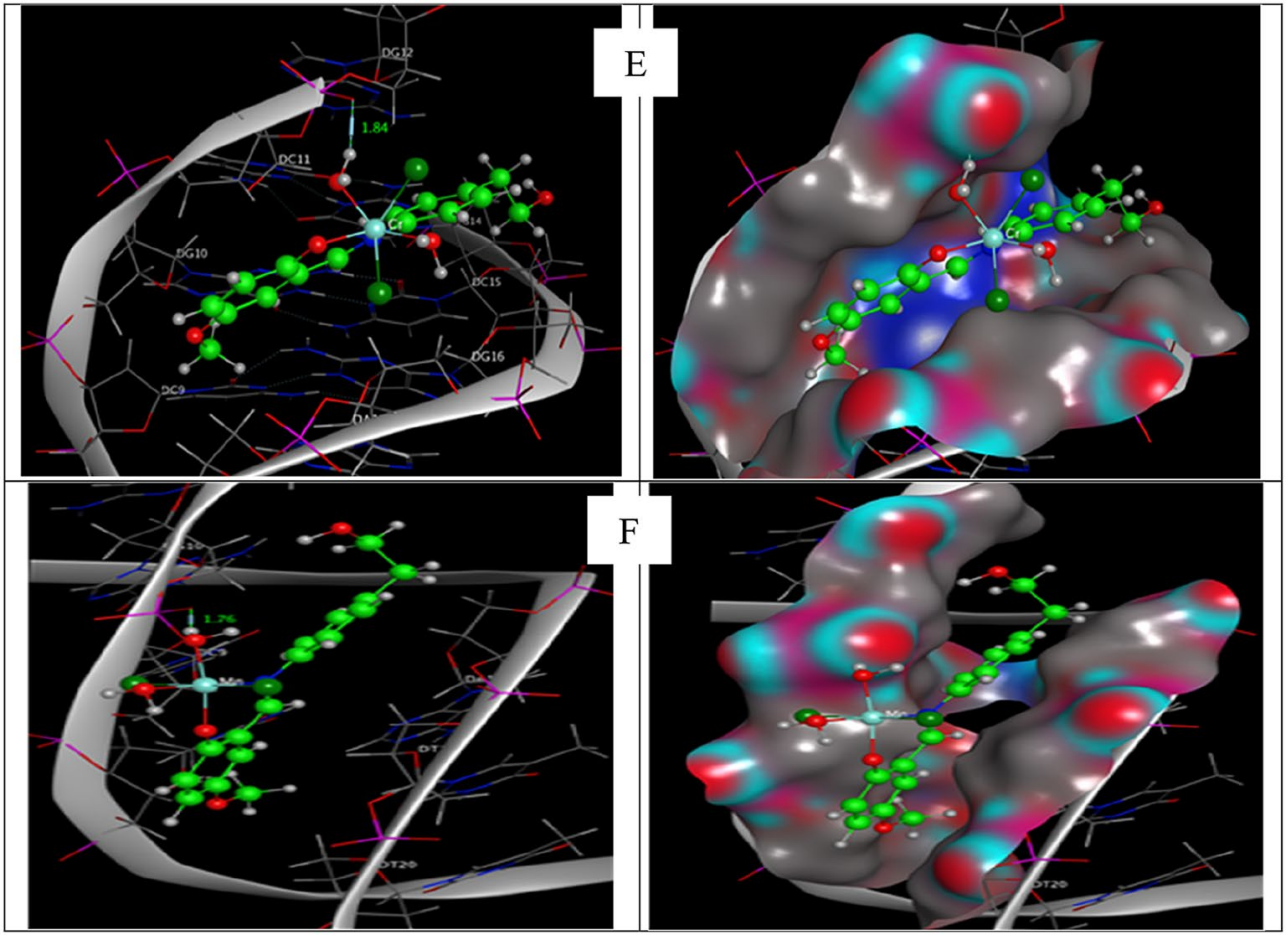
Molecular docking studies showed the interaction of HL1 and HL2 with the active site of the human DNA receptor, as well as with [Fe(L1)(H_2_O)_2_*Cl*_2_](C), [Cr(L1)(H_2_O)_2_*Cl*_2_](D), [Cr(L2)(H_2_O)_2_*Cl*_2_] (E) and [Mn(L2)(H_2_O)_3_*Cl*](F)** (**PDB ID:1BNA).

Molecular docking has developed into a crucial tool in drug discovery since the first algorithms were created in the 1980s^42,43^. This is due to its capacity to accurately anticipate the conformation of small-molecule ligands inside suitable target binding sites^44,45^. The newly synthesized complexes were submitted to computational docking investigations in the molecular docking study to comprehend their interactions with DNA and explore the probable binding mode^46^. Figures 14 and 15 show the energy-minimized docked poses for the HL1 ligand and its CrL1, FeL1, and HL2 and its CrL2 and MnL2 complexes, which show that the three complexes' ideal potential conformation lies inside the DNA base pairs. The outcomes of the molecular docking can be used to integrate the three complexes into the DNA molecule to halt DNA replication. The outcomes of electronic testing can be further validated using molecular docking investigations.

The major active site of the human DNA receptor (PDB ID: 1BNA), Table 11, and Figs. 14 and 15 were molecular docked using the MOA2014 program to determine potential modes of binding.

**Table 11:** The docking interaction data computations of HL1, HL2, [Fe(L1)(H_2_O)_2_*Cl*_2_], [Cr(L1)(H_2_O)_2_*Cl*_2_], [Cr(L2)(H_2_O)_2_*Cl*_2_] and [Mn(L2)(H_2_O)_3_*Cl*] with the active sites of the receptor of human DNA (PDB ID:1BNA).

Compounds	Receptor	Interaction	Distance(Å)*	E (kcal/mol)
HL1				
O_3_	OP1 DG 10 (A)	H-donor	3.18 (2.22)	− 1.7
6-ring	C5’ DG 12 (A)	pi-H	4.71	− 0.6
HL2				
O_12_	OP1 DG 10 (A)	H-donor	3.05 (2.09)	− 2.2
[Fe(L1)(H_2_O)_2_*Cl*_2_]				
O_33_	OP1 DG 10 (A)	H-donor	2.68 (1.72)	− 22.0
O_33_	OP1 DG 10 (A)	ionic	2.68	− 7.0
O_34_	OP1 DG 10 (A)	ionic	3.13	− 3.6
[Cr(L1)(H_2_O)_2_*Cl*_2_]				
C_11_	OP1 DG 12 (A)	H-donor	3.77	− 0.7
O_33_	O3’ DA 17 (B)	H-donor	2.92	− 3.3
O_33_	OP1 DA 18 (B)	H-donor	2.74	− 16.7
O_34_	OP1 DA 18 (B)	H-donor	3.19	− 5.9
O_33_	OP1 DA 18 (B)	ionic	2.74	− 6.5
O_34_	OP1 DA 18 (B)	ionic	3.19	− 3.3
6-ring	C4’ DA 17 (B)	pi-H	4.15	− 0.6
[Cr(L2)(H_2_O)_2_*Cl*_2_]				
O_33_	OP1 DG 12 (A)	H-donor	2.80 (1.84)	− 20.8
O_33_	OP1 DG 12 (A)	ionic	2.80	− 5.9
6-ring	C5’ DG 12 (A)	pi-H	3.99	− 1.0
[Mn(L2)(H_2_O)_3_*Cl*]				
O_33_	OP1 DG 10 (A)	H-donor	2.74 (1.76)	− 21.9
N_10_	OP1 DG 10 (A)	ionic	3.62	− 1.5
O_33_	OP1 DG 10 (A)	ionic	2.74	− 6.5

*The lengths of H-bonds are in brackets.

The output of the Gaussian 09 software was used to build the PDB file format structure of the ligands and complexes. You may obtain the crystal structures of the human DNA receptor (PDB ID: 1BNA) from the protein data bank at http://www.rcsb.org/pdb.

The binding free energies of HL1, HL2, and their metal complexes with the protein receptor (PDB:1bna) are determined to be − 2.3, − 2.2, − 27.0, − 27.7, − 32.6, and − 29.9 kcal/mol in the current study for HL1, HL2, [Cr(L1)(H_2_O)_2_*Cl*_2_], [Cr(L_2_)(H_2_O)_2_*Cl*_2_], [Fe(L1)(H_2_O)_2_*Cl*_2_] and [Mn(L2)(H_2_O)_3_*Cl*] complexes; respectively, Table 11. Stronger interactions result from binding energies that are more negative. The interaction is therefore of the kind [Fe(L1)(H_2_O)_2_*Cl*_2_]˃[Mn(L2)(H_2_O)_3_*Cl*]˃[Cr(L_2_)(H_2_O)_2_*Cl*_2_]˃[Cr(L1)(H_2_O)_2_*Cl*_2_] > L_1_ > L_2_.

These results are in excellent agreement with the experimental results from the spectroscopic measurement of the Kb values.

#### In silico ADMET analysis of ligands and their complexes

ADMET (Absorption, Distribution, Metabolism, Elimination, Toxicity) prediction is used to predict the toxicity and pharmacological properties of studied compounds using the Swiss ADME server, Tables 12 and 13.

**Table 12:** ADME analysis of the HL1 and its complexes.

ADME feature^a^	HL1	CrL1	FeL1
Physicochemical properties			
Formula	C_15_H_14_BrNO_2_	C_15_H_18_BrCl_2_CrNO_4_	C_15_H_18_BrCl_2_FeNO_4_
Fraction Csp3	0.13	0.20	0.20
Num. H-bond acceptors	3	3	4
Num. H-bond donors	2	4	3
Molar refractivity	80.80	99.66	99.66
TPSA	52.82 Å^2^	51.16 Å^2^	51.16 Å^2^
Lipophilicity			
Log *P*_o/w_ (iLOGP)	2.59	0.00	0.00
Log *P*_o/w_ (XLOGP3)	3.13	3.64	3.64
Log *P*_o/w_ (WLOGP)	3.44	3.94	3.94
Log *P*_o/w_ (MLOGP)	2.88	0.83	0.83
Log *P*_o/w_ (SILICOS-IT)	4.27	− 0.72	− 0.70
Consensus Log *P*_o/w_	3.26	1.54	1.54
Water solubility			
Log *S* (ESOL)	− 4.00	− 5.09	− 5.11
Solubility	3.20e–02 mg/ml; 9.99e–05 mol/l	3.89e–03 mg/ml; 8.12e–06 mol/l	3.71e–03 mg/ml; 7.68e–06 mol/l
Class	Moderately soluble	Moderately soluble	Moderately soluble
Log *S* (Ali)	− 3.91	− 4.40	− 4.40
Solubility	3.95e–02 mg/ml; 1.23e–04 mol/l	1.90e–02 mg/ml; 3.96e–05 mol/l	1.91e–02 mg/ml; 3.96e–05 mol/l
Class	Soluble	Moderately soluble	Moderately soluble
Log *S* (SILICOS-IT)	− 5.50	− 4.69	− 4.70
Solubility	1.02e–03 mg/ml; 3.18e–06 mol/l	9.76e–03 mg/ml; 2.04e–05 mol/l	9.64e–03 mg/ml; 2.00e–05 mol/l
Class	Moderately soluble	Moderately soluble	Moderately soluble
Pharmacokinetics			
GI absorption	High	High	High
BBB permeant	Yes	Yes	Yes
P-gp substrate	No	Yes	Yes
CYP1A2 inhibitor	Yes	No	No
CYP2C19 inhibitor	Yes	No	No
CYP2C9 inhibitor	Yes	No	No
CYP2D6 inhibitor	Yes	No	No
CYP3A4 inhibitor	Yes	Yes	Yes
Log *K*_p_ (skin permeation)	− 6.03 cm/s	− 6.64 cm/s	− 6.66 cm/s
Medicinal chemistry			
PAINS	0 alert	0 alert	0 alert
Brenk	1 alert: imine_1	0 alert	1 alert: heavy_metal
Synthetic accessibility	2.57	4.67	4.81

**Table 13:** ADME analysis of the HL2 and its complexes.

ADME feature^a^	HL2	CrL2	MnL2
Physicochemical properties			
Formula	C_16_H_17_NO_3_	C_16_H_21_Cl_2_CrNO_5_	C_16_H_23_ClMnNO_6_
Fraction Csp3	0.19	0.25	0.25
Num. H-bond acceptors	4	5	6
Num. H-bond donors	2	3	4
Molar refractivity	79.52	97.68	94.88
TPSA	62.05 Å^2^	60.39 Å^2^	69.62 Å^2^
Lipophilicity			
Log *P*_o/w_ (iLOGP)	2.63	0.00	0.00
Log *P*_o/w_ (XLOGP3)	2.41	2.92	1.64
Log *P*_o/w_ (WLOGP)	2.69	3.20	2.44
Log *P*_o/w_ (MLOGP)	1.91	− 0.10	− 1.14
Log *P*_o/w_ (SILICOS-IT)	3.64	− 1.35	− 3.13
Consensus Log *P*_o/w_	2.66	0.93	− 0.04
Water solubility			
Log *S* (ESOL)	− 3.15	− 4.26	− 3.36
Solubility	1.90e–01 mg/ml; 7.01e–04 mol/l	2.36e–02 mg/ml; 5.49e–05 mol/l	1.80e–01 mg/ml; 4.32e–04 mol/l
Class	Soluble	Moderately soluble	Soluble
Log *S* (Ali)	− 3.36	− 3.85	− 2.72
Solubility	1.20e–01 mg/ml; 4.41e–04 mol/l	6.08e–02 mg/ml; 1.41e-–04 mol/l	8.01e–01 mg/ml; 1.93e–03 mol/l
Class	Soluble	Soluble	Soluble
Log *S* (SILICOS-IT)	− 4.79	− 4.02	− 3.48
Solubility	4.35e–03 mg/ml; 1.60e–05 mol/l	4.15e–02 mg/ml; 9.64e–05 mol/l	1.37e–01 mg/ml; 3.29e–04 mol/l
Class	Moderately soluble	Moderately soluble	Soluble
Pharmacokinetics			
GI absorption	High	High	High
BBB permeant	Yes	Yes	Yes
P-gp substrate	No	Yes	Yes
CYP1A2 inhibitor	Yes	No	No
CYP2C19 inhibitor	No	No	No
CYP2C9 inhibitor	No	No	No
CYP2D6 inhibitor	Yes	No	No
CYP3A4 inhibitor	Yes	Yes	No
Log *K*_p_ (skin permeation)	− 6.24 cm/s	− 6.85 cm/s	− 7.67 cm/s
Medicinal chemistry			
PAINS	0 alert	0 alert	0 alert
Brenk	1 alert: imine_1	0 alert	0 alert
Synthetic accessibility	2.59	4.86	4.90

^a^*TPSA* topological polar surface area, Consensus LogP_o/w_: Average of all five predictions, GI absorption: Gastrointestinal absorption, P-gp substrate: P-glycoprotein substrate, CYP1A2 inhibitor: Cytochrome P450 1A2 inhibitor, CYP2C19 inhibitor: Cytochrome P450 2C19 inhibitor, CYP2C9 inhibitor: Cytochrome P450 2C9 inhibitor, CYP2D6 inhibitor: Cytochrome P450 2D6 inhibitor, CYP3A4 inhibitor: Cytochrome P450 3A4 inhibitor, PAINS: Pan Assay Interference Structures, Brenk: Structural Alert, Synthetic accessibility score: from 1 (very easy) to 10 (very difficult).

Most lead-like compounds fail in clinical trials due to weak pharmacokinetic ADMET properties. Before lead-like compounds enter the preclinical phases, in silico testing aids in drug design development, saving time and resources. Using SWISS ADME online software, the pharmacokinetic behavior of the Schiff base ligands and their complexes was investigated. Using Lipinski’s rule of five, the software's output may be assessed, and the results are shown in Tables 12 and 13^47^. Because of their computed log p values of 3.26, 1.54, and 1.54 for HL1, CrL1, and FeL1 respectively, the ligand HL1 and its CrL1 and FeL1 complexes may permeate biological membranes. According to SWISS ADME software, the CrL2 and MnL2 of the HL2 ligand have respective values of 2.66, 0.93, and 0.04 (less than 5) respectively. Total polar surface area (TPSA) values for the ligands HL1, CrL1, and FeL1 are 52.82, 51.16, and 51.16 2 respectively (below 140 2). The hydrogen bond acceptors are 3, 3, 4 while the hydrogen bond donors for HL1, CrL1, and FeL1 are 2, 4, 3. (which are also less than 5 and 10)^48^. Additionally, the hydrogen bond donors are 2, 3, and 4, while the acceptors are 4, 5, and 6 for the ligand HL2 and its complexes CrL2 and MnL2. The ligand HL2 and its complexes CrL2, and MnL2 have 62.05, 60.39, and 69.62 2 Å^2^ each (below 140 Å^2^), respectively. These data imply the efficient transport of it by oral method and inside the GIT (Gastrointestinal Tract) and BBB (Blood Brain Barrier). The bioavailability score is 0.55 (> than 0) which also reveals that the Schiff bases and their complexes are biologically active. Similarly, the ADMET property analysis reveals that it has a 95.64% ability to undergo absorption inside the human GIT. The log Kp value of the compounds.

HL1, CrL1, and FeL1 are − 6.03, − 6.64, and − 6.66 cm/s and − 6.24, − 6.85, and − 7.67 cm/s for compounds HL2, CrL2, and MnL2 respectively (which is less than 2.5). The results show that the ligands and their complexes are distributed more in blood plasma and less in tissue and therefore no renal failure and dehydration is caused. It is metabolized by CYP1A2 inhibitor, CYP2C19 inhibitor, CYP2C9 inhibitor, CYP2D6 inhibitor, and CYP3A4 inhibitor enzymes. These enzymes render the oxidation process and facilitate their excretion. It has nontoxic value and non–carcinogen in nature.

#### Structure–activity relationship (SAR)

The practical activity ranking cannot be determined by only one parameter, it depends on a combination of parameters^49,50^. Thus, there is an urgent need to derive the Structure–Activity Relationship (SAR) model to correlate the practical activity of the subject compounds (herein IC_50_) with their calculated chemical descriptors and find the most effective description model^51,52^. SAR model was constructed by correlating the practical activity (IC_50_) of the subject compounds with their theoretical chemical descriptors (E_HOMO_, E_LUMO_, ΔE, IP, EA, χ, µ, η, σ, ω and Nu), Eq. (4)^53,54^.

4 $${\text{IC}}_{{{5}0}} = {\text{constant}} + {\mathbf{a}}_{{\mathbf{1}}} {\text{E}}_{{{\text{HOMO}}}} + {\mathbf{a}}_{{\mathbf{2}}} {\text{E}}_{{{\text{LUMO}}}} + {\mathbf{a}}_{{\mathbf{3}}} \Delta {\text{E}} + {\mathbf{a}}_{{\mathbf{4}}} \chi + {\mathbf{a}}_{{\mathbf{5}}} {\text{CP}} + {\mathbf{a}}_{{\mathbf{6}}} \eta + {\mathbf{a}}_{{\mathbf{7}}} \sigma + {\mathbf{a}}_{{\mathbf{8}}} \omega + {\mathbf{a}}_{{\mathbf{9}}} {\text{Nu}} + {\mathbf{a}}_{{{\mathbf{10}}}} \mu$$

where; E_HOMO_ is the HOMO energy, E_LUMO_ is the LUMIO energy, ΔE is the HOMO–LUMO energy gap, IP is the ionization energy, EA is the electron affinity, χ is the electronegativity, CP is the chemical potential, η is the chemical hardness, σ is the chemical softness, ω is the electrophilicity index, Nu is the nucleophilicity index, µ is the dipole moment. These parameters were calculated as previously reported^55,56^.

The theoretical SAR formula was derived for practical activity (IC_50_), Eqs. (5, 6, 7, 8);

5 $${\text{IC}}_{{{5}0}} \;\left( {{\text{for }}\;{\text{L1 }}\;{\text{and }}\;{\text{its }}\;{\text{complexes}}\;{\text{ against }}\;{\text{MCF}} - {7}} \right) = {89}.{35} - {5}.{13}\omega + {2}.00\mu$$

6 $${\text{IC}}_{{{5}0}} \left( {{\text{for}}\;{\text{ L1}}\;{\text{ and}}\;{\text{ its}}\;{\text{ complexes}}\;{\text{ against }}\;{\text{MCF}} - {7}} \right) = {89}.{35} - {5}.{13}\omega + {2}.00\mu$$

7 $${\text{IC}}_{{{5}0}} \;\left( {{\text{for }}\;{\text{L1}}\;{\text{ and }}\;{\text{its }}\;{\text{complexes}}\;{\text{ against }}\;{\text{HepG2}} - {2}} \right) = {11}0.{24} - {5}.{75}\omega - {12}.{35}\mu$$

8 $${\text{IC}}_{{{5}0}} \;\left( {{\text{for}}\;{\text{ L2 }}\;{\text{and}}\;{\text{ its }}\;{\text{complexes}}\;{\text{ against}}\;{\text{ HepG2}}} \right) = - {215}.{25} + {45}.{8}0{\text{IP}} + {1}.{31 }\omega$$

From Eqs. (2 3, 4, 5), it is obvious that practical activity depends on both of electrophilicity index (ω) and dipole moment (µ) in the case of L_1_ and its complexes against both of MCF-7 and HepG2. While, practical activity depends on both of Ionization potential (IP) and electrophilicity index (ω) in the case of L_2_ and its complexes against both of MCF-7 and HepG2. To investigation the validity of derived formula, IC_50_ values were calculated using Eqs. (5, 6, 7, 8) and compared with practical values, Table 14, which showed excellent agreement.

**Table 14:** Structure–activity relationship (SAR) model for practical and calculated IC_50_ values in μM for anticancer activity against MCF-7 and HepG2.

	MCF-7		HepG2	
	IC_50_ (Practical)	IC_50_ (Calc.)	IC_50_ (Practical)	IC_50_ (Calc.)
HL1	70.000	70.049	75.000	74.979
CrL1	37.000	37.062	21.000	20.957
FeL1	60.000	60.061	29.000	28.972
HL2	69.000	68.780	52.000	51.964
CrL2	37.000	36.790	41.000	40.899
MnL2	3.000	2.816	2.600	2.569

## Conclusion

In conclusion, our work concentrated on four novel complexes of Fe(III), Mn(II), and Cr(III) obtained from two bidentate ligands, each of which had a different substituent in the salicylaldimine molecule. Various spectroscopic and analytical approaches were used to suggest the chemical structures of the novel complexes FeL1, CrL1, CrL2, and MnL2. The non-electrolytic nature of the compounds was concluded based on the molar conductivity results. FT-IR spectral data revealed that the two ligands HL1 and HL2 behave as monobasic bidentate ligands coordinating the Fe(III), Cr(III), Mn(II) metal ions through the phenolic OH and C = N functional groups. UV–vis spectral data and magnetic moment values suggest the octahedral structure of all complexes, the octahedral geometry of the novel compounds has been validated and optimized by DFT analysis. The estimated parameters showed that the free ligands provide electrons to the various metal ions, establishing a stable six-membered chelate ring. All the novel complexes have shown enhanced antibacterial efficacy against a wide spectrum of microorganisms. The in vitro cytotoxicity results showed that the new compounds were significantly more hazardous to cancer cells than the widely used drug cisplatin, which has an IC_50_ of 4.0 µM and 4.8 µM against the Hep-G2 and MCF-7 cell lines respectively. MnL2 complex has superior anticancer efficacy compared to the other complexes. Molecular docking study confirmed the binding affinity of the complexes with DNA, the obtained results revealed that the binding energy of the free ligands and their complexes with the receptor PDB:1bna follows the order: FeL1 > MnL2 > CrL2 > CrL1 > HL1 > HL2. FeL1 and MnL2 complexes showed superior binding energy values of − 22.0 and − 21.9 kcal/mol. The pharmacokinetic and biological actions of the two Schiff bases and their complexes were predicted using Swiss ADME, PASS and pkCSM online softwares.

## Supplementary Information

Supplementary Information 1.

Supplementary Information 2.

Supplementary Information 3.

## Data Availability

The data supporting the findings of this study are available in the supplementary material of this article.
