# Supplementary figures and images for: Novel Bromo and methoxy substituted Schiff base complexes of Mn(II), Fe(III), and Cr(III) for anticancer, antimicrobial, docking, and ADMET studies
Source: Sci Rep. 2023 Feb 23;13:3199. doi: 10.1038/s41598-023-29386-2 (PMC9950075; doi:10.1038/s41598-023-29386-2)

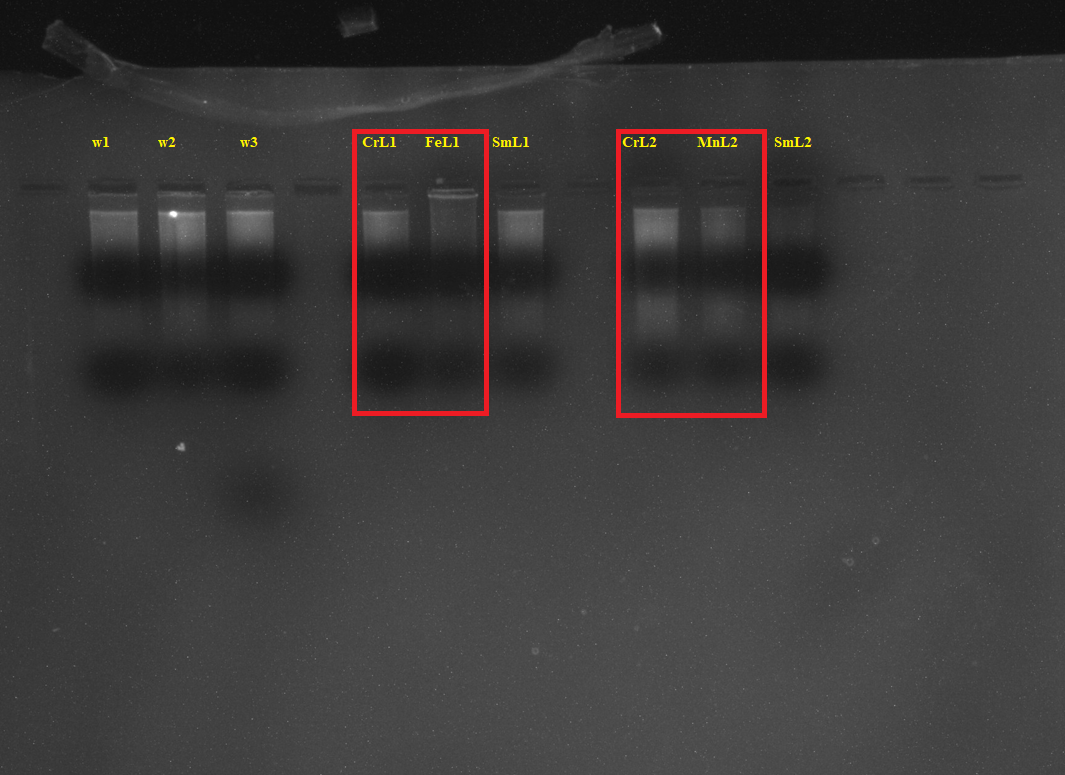

Supplement: Supplementary file 2 — Supplementary Information 2. [file 41598_2023_29386_MOESM2_ESM.png]
