# Supplementary material for: Novel Bromo and methoxy substituted Schiff base complexes of Mn(II), Fe(III), and Cr(III) for anticancer, antimicrobial, docking, and ADMET studies
Source: Sci Rep. 2023 Feb 23;13:3199. doi: 10.1038/s41598-023-29386-2 (PMC9950075; doi:10.1038/s41598-023-29386-2)
Supplement: Supplementary file 3 — Supplementary Information 3. [file 41598_2023_29386_MOESM3_ESM.docx]

**Novel Bromo and methoxy substituted Schiff base complexes of Mn(II), Fe(III), and Cr(III) for anticancer, antimicrobial, docking, and ADMET studies**

Laila H. Abdel-Rahman*^a^, Amani A. Abdelghani ^b, c^ Abeer A. AlObaid ^d^, Doaa Abou El-ezz ^e^, Ismail Warad ^f^, Mohamed R Shehata ^g^, and Ehab M. Abdalla ^h^

^a^**^*^** Chemistry Department, Faculty of Science, Sohag University, 82534 Sohag, Egypt

^b^ Department of Chemistry University of Prince Edward Island 550 University Avenue, Charlottetown, PE C1A 4P3, Canada

^c^ Chemistry Department, Faculty of Science, Damanhour University, Damanhour 22511, Egypt

^d^ Department of Chemistry, College of Science, King Saud University, Riyadh-11451, Saudi Arabia

^e^ Department of Pharmacology and Toxicology, Faculty of Pharmacy, October University for Modern Sciences and Arts (MSA University), Cairo, Egypt

^f^ Department of Chemistry, AN-Najah National University, P.O. Box 7, Nablus, Palestine

^g^ Chemistry Department, Faculty of Science, Cairo University, Giza, Egypt

^h^ Chemistry Department, Faculty of Science, New Valley University, Alkharga 72511, Egypt

**Section S1: *Physical measurements***

A CHNS-932 (LECO) Varo elemental analyzer was used to do microanalyses of carbon, hydrogen, and nitrogen at the Microanalytical Center, Cairo University, Egypt. Shimadzu FTIR spectrophotometer (model 8101) 400–4000 cm^−1^ Fourier transform infrared (FT-IR) spectra were captured in KBr pellets. Tetra methyl silane (TMS) was utilized as an internal standard while 1H and 13C NMR spectra were captured in DMSO-d6 using a Bruker 400 MHz spectrometer. An HP MS-5988 GS-MS was used to record mass spectra at 70 eV using the electron ionization technique. With the use of a Shimadzu UV min i- 1240 spectrophotometer, UV-visible spectra were collected. A Jenway 4010 conductivity meter was used to test the molar conductivities of 10^-3^ M solutions of the solid complexes in DMF.

Using a Shimadzu TG-50H thermal analyzer, thermogravimetric (TG) and differential thermogravimetric (DTG) studies of the solid complexes were performed from an ambient temperature to 1000°C. At Cairo University at Egypt's Microanalytical Center, an antimicrobial bioassay was conducted. At the National Cancer Institute, the Cancer Biology Department, and the Pharmacology Department at Cairo University, anticancer activity experiments were conducted.

**Section S2. Antimicrobial screening studies**

Each compound was given a 1 percent standard solution, and 1 ml of the solution was diluted with 9 ml of DMSO solvent. Petri dishes of an equivalent diameter were sterilized at 180°C. Four different concentrations of each compound's stock solution were made: 10, 20, 50, and 100 g/ml. One mL of each concentration solution was added to nine mL of agar medium on pre-sterilized Petri dishes.

After that, bacterial and fungal strains were added to the dishes (diameter 5 mm). The dishes were injected at 30 2 °C. The investigated organism's colony diameter was measured after six days using a millimeter scale. The diameter of the inhibition zone was measured in millimeters (mm) and contrasted with the gentamicin and ketoconazole growth inhibition standards. DMSO was employed as a control under identical circumstances as the organisms, and no activity was found. Three copies of the operation's findings were made. The formula used to calculate the percentage of inhibition of the test organism's development is (Cd -Td) / Cd x 100 = percent inhibition, where Cd is the colony diameter of the control set and Td is the colony diameter of the treated set.

**Section S3. Cytotoxicity**

The MTT assay was then carried out after the plate spent 4 hours in a CO_2_ incubator at 37 °C. The test is designed to identify metabolically active cells by measuring the amount of insoluble purple formazan that is produced when metabolically active cells convert the tetrazolium salt MTT to this color. The IC_50_ was defined as the concentration that would prevent 50% of the cell growth compared to untreated cells. IC_50_ values were calculated using Chart Pad Prism version 6.01 from 2012. (GraphPad Software, San Diego, USA). It is possible to create a dose-effect curve by graphing the log concentration against the matching viability percent.

**Table S1.**Antibacterial activity of the Schiff base ligands HL1 and HL2 and their Cr(III), Fe(III) and Mn(II) complexes

| **Compounds** | ***S. aureus*** *(+ve)* | ***B. subtilis*** *(+ve)* | ***E. coli*** *(-ve)* | ***P. vulgaris* RCMB** *(-ve)* |
| --- | --- | --- | --- | --- |
| **HL1** | 7 ± 0.24 | 8 ± 0.18 | 2 ± 0.08 | 1 ± 0.21 |
| **HL2** | 12 ± 0.29 | 13 ± 0.21 | 10 ± 0.20 | 12 ± 0.25 |
| **CrL1** | 17 ± 0.31 | 18 ± 0.19 | 7± 0.13 | 20 ± 0.14 |
| **FeL1** | 14 ± 0.18 | 17± 0.12 | 5 ± 0.06 | 16 ± 0.14 |
| **CrL2** | 15 ± 0.22 | 15± 0.16 | 14 ± 0.50 | 13± 0.30 |
| **MnL2** | 16 ± 0.28 | 18± 0.19 | 13 ± 0.20 | 22± 0.12 |
| **Gentamycin** | 24 ± 0.51 | 26 ± 0.31 | 30 ± 0.23 | 25 ± 0.30 |

**Table S2.**Antifungal activity of the Schiff base ligands HL1 and HL2 and their Cr(III), Fe(III) and Mn(II) complexes

| **Compounds** | ***A. Fumigates*** | ***C. Albicans*** |
| --- | --- | --- |
| **HL1** | 5 ± 0.12 | 8 ± 0.14 |
| **HL2** | 15 ± 0.16 | 19 ± 0.20 |
| **CrL1** | NA | NA |
| **FeL1** | NA | NA |
| **CrL2** | NA | NA |
| **MnL2** | NA | NA |
| **Ketoconazole** | 20 ± 0.28 | 24 ± 0.30 |
| **DMSO** | 0.00 | 0.00 |

**Table S3:** Cytotoxic activity (IC_50_) of the HL1 and HL2 ligands and their Cr(III), Fe(III), Mn(II) complexes against Breast carcinoma cells, (MCF-7 cell line) and hepatic cellular carcinoma cells, (HepG-2) with reference to Cisplatin standard drug.

| Compounds | IC_50_(µg/µl) | |
| --- | --- | --- |
|  | MCF-7 | HepG2 |
| HL1 | 70.00 | 75.00 |
| CrL1 | 37.00 | 21.00 |
| FeL1 | 60.00 | 29.00 |
| HL2 | 69.00 | 52.00 |
| CrL2 | 37.00 | 41.00 |
| MnL2 | 3.00 | 2.60 |
| Cisplatin | 4.80 | 4.00 |


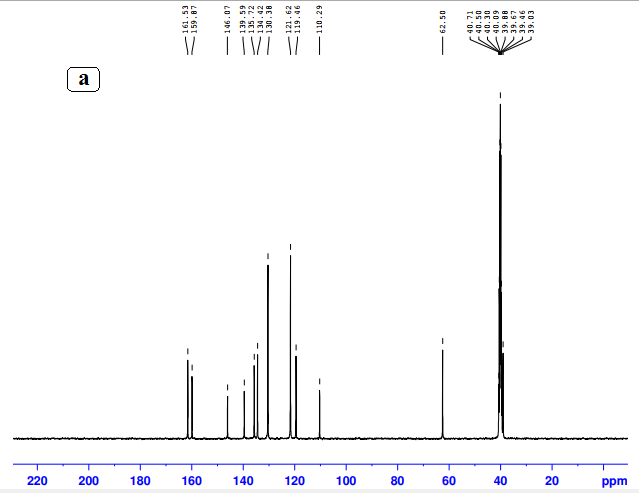

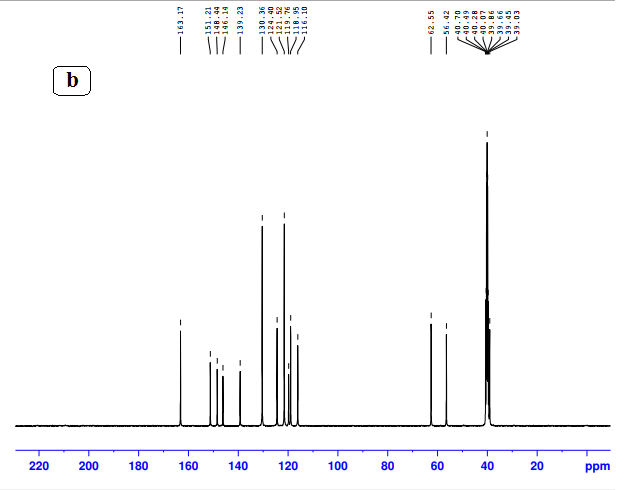


**Figure S1:**^13^CNMR spectra of HL1 (a) and HL2 ligands (b).

**
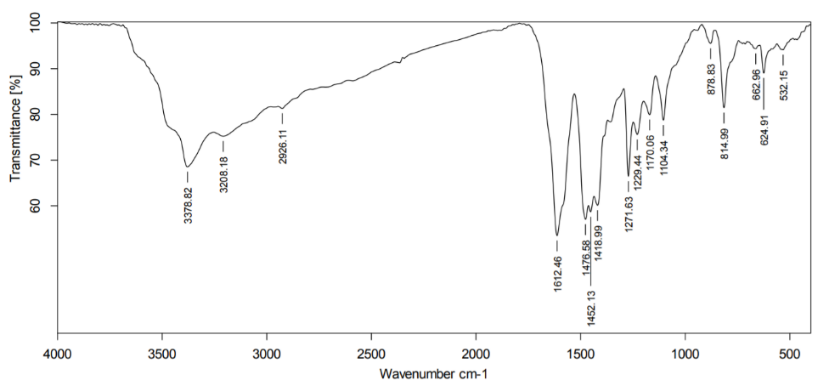
**

HL1

**
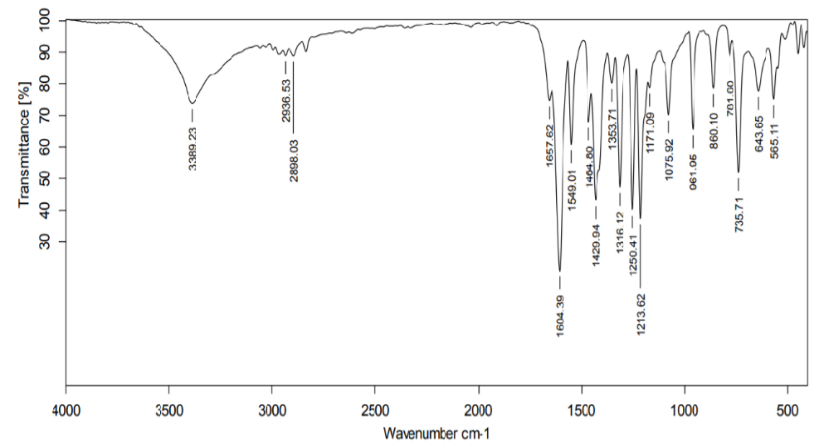
**

FeL1

**
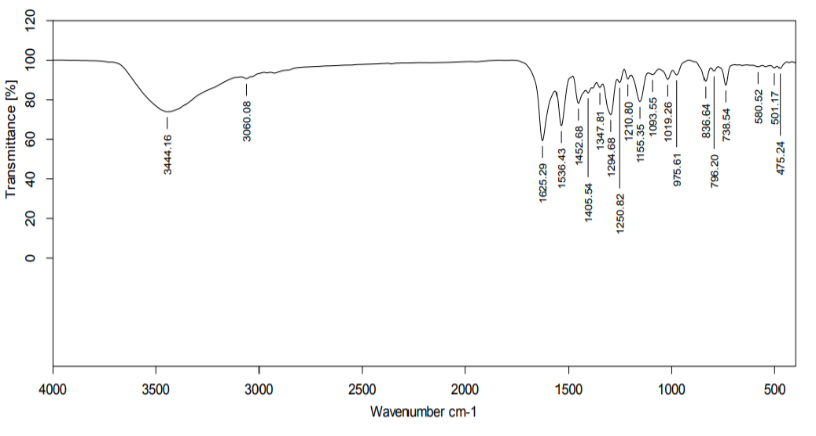
**

Cr L1

**
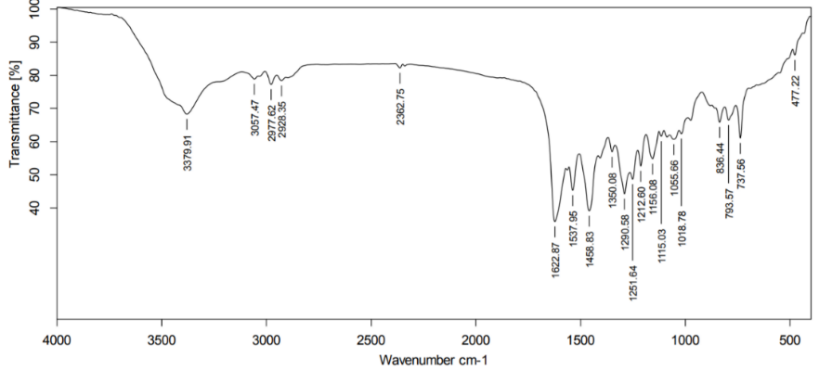
**

HL_2_

**
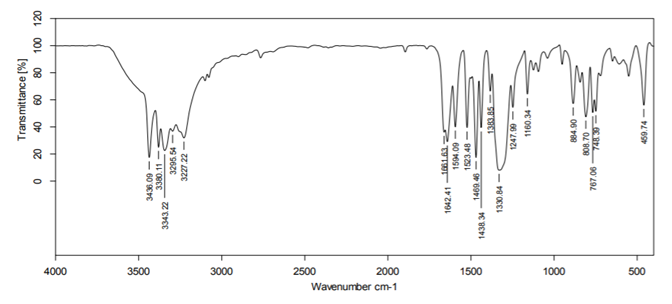
**

CrL_2_

**
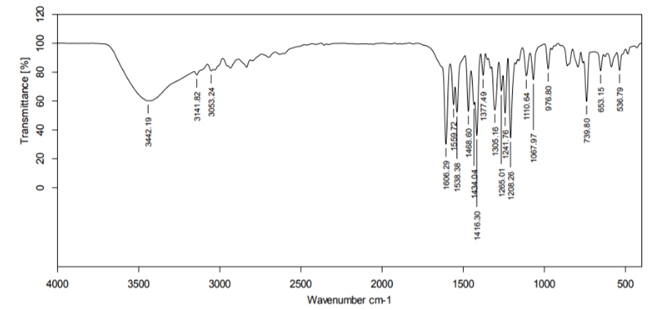
**

Mn L_2_

**Figure S2**: Infrared spectral of the new HL1 and HL2 ligands and their new Fe(III),

Mn(II) and Cr(III) complexes at 4000 - 400 cm ^-1^.


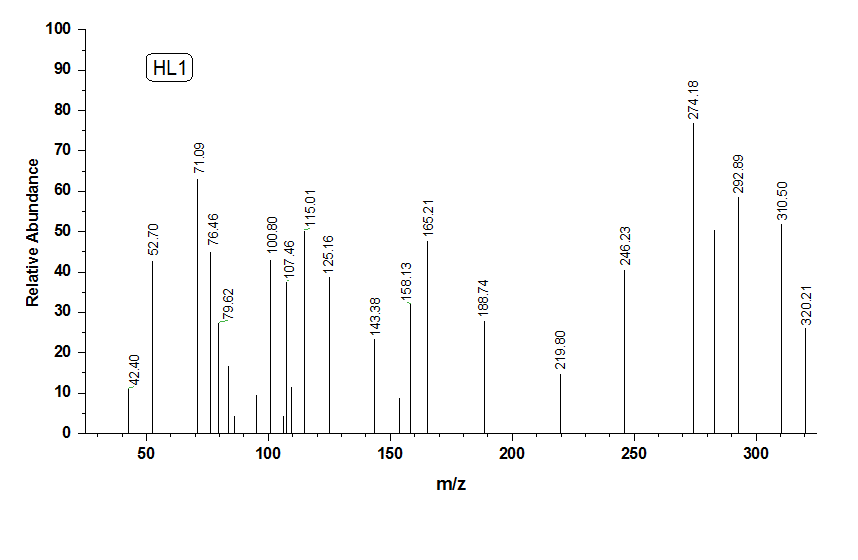


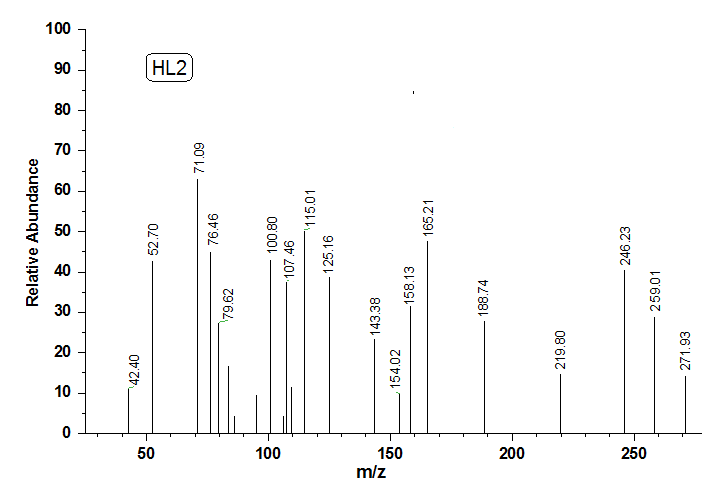


**Figure S3:** Mass spectra of HL1 and HL2 ligands in DMF with concentration 10^-3^ M at 298 K.


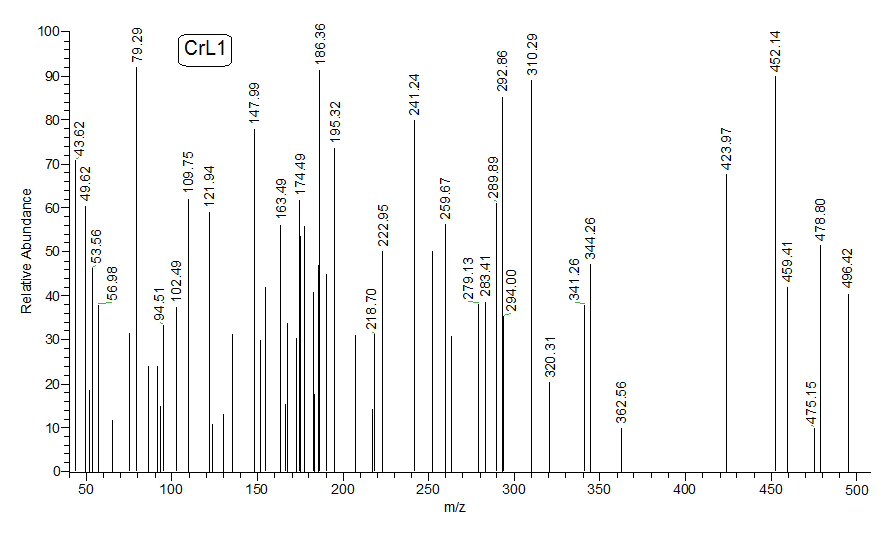


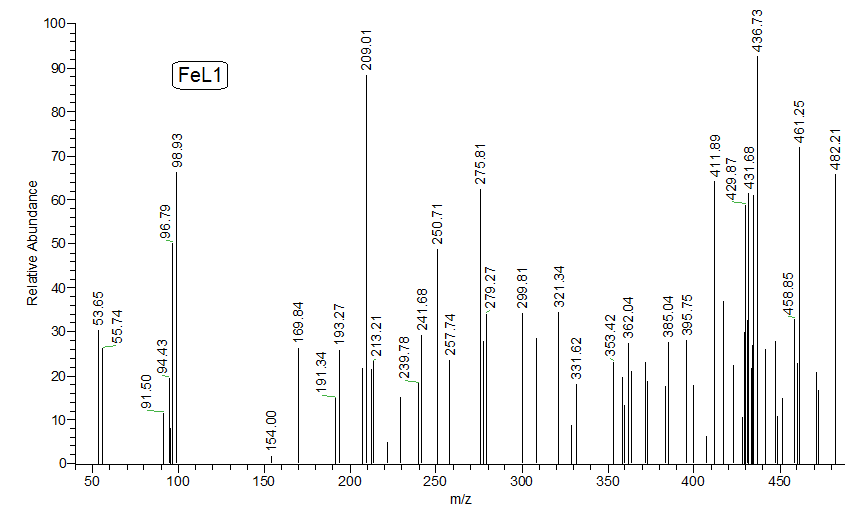


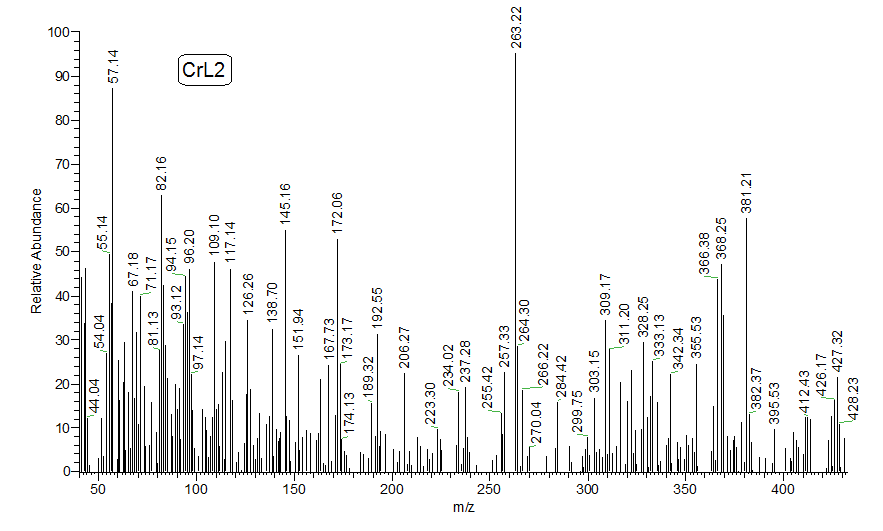


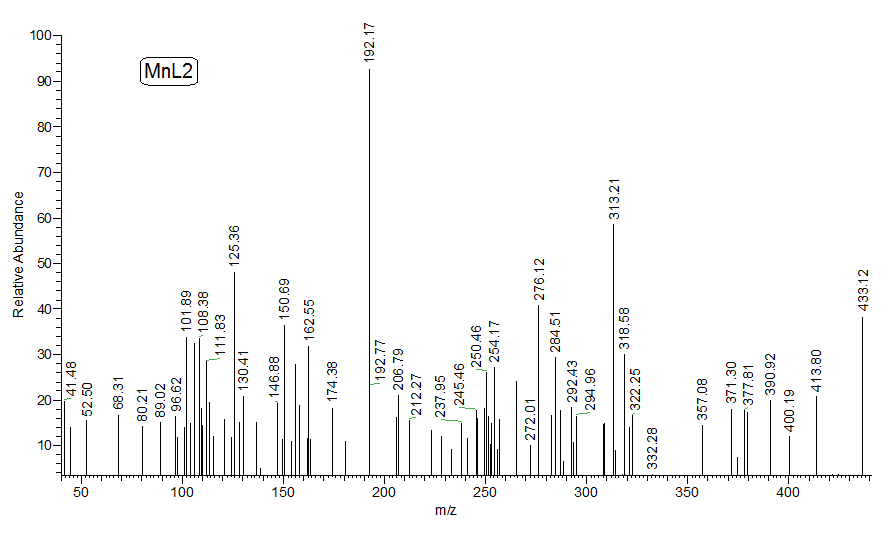


**Figure S4**: Mass spectra of CrL1, FeL1, CrL2 and MnL2 complexes in DMF with a concentration of 10^-3^ M at 298 K.


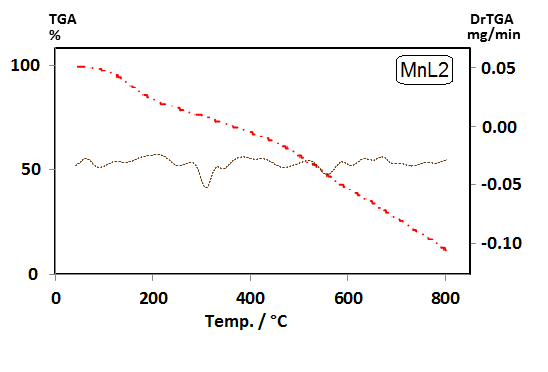


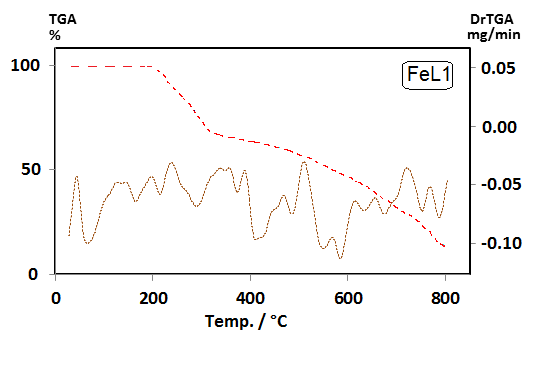


**Figure S5:** TGA and DTG curve of FeL1 and MnL2 complex from ambient temperature to 1000 ºC at a heating rate of 10 degrees/min. in air atmosphere

**Figure S6: (a)** UV-vis spectra of FeL1 and CrL1 in Tris buffer (pH 7.5, 298 K) and in the absence and the presence of CT-DNA, the complex concentration was 1x10^-3^ M^-1^ and CT-DNA concentrations were 10-100 μ M corresponding to the curves from 1 to 10, respectively
